# Interaction of Signaling Lymphocytic Activation Molecule Family 1 (SLAMF1) receptor with *Trypanosoma cruzi* is strain-dependent and affects NADPH oxidase expression and activity

**DOI:** 10.1371/journal.pntd.0008608

**Published:** 2020-09-14

**Authors:** Cristina Poveda, Alfonso Herreros-Cabello, Francisco Callejas-Hernández, Jesús Osuna-Pérez, María C. Maza, Carlos Chillón-Marinas, Jossela Calderón, Konstantinos Stamatakis, Manuel Fresno, Núria Gironès

**Affiliations:** 1 Centro de Biología Molecular Severo Ochoa, Consejo Superior de Investigaciones Científicas, Universidad Autónoma de Madrid, Cantoblanco, Madrid, Spain; 2 Instituto Sanitario de Investigación Princesa, Madrid, Spain; Universiteit Antwerpen, BELGIUM

## Abstract

The receptor Signaling Lymphocyte-Activation Molecule Family 1 (SLAMF1) controls susceptibility to Infection by the lethal *Trypanosoma cruzi* Y strain. To elucidate whether genetic diversity of the parasite was related with disease susceptibility, we further analyzed the role of SLAMF1 using 6 different *Trypanosoma cruzi* strains including Y. The interaction of SLAMF1 receptor with *T*. *cruzi* was evidenced by fluorescence microscopy, flow cytometry and quantitative PCR. All the strains, except VFRA, showed a decrease in parasite load in infected macrophages in *Slamf1*^*-/-*^ compared to BALB/c. In macrophages gene expression NADPH oxidase (NOX2), and reactive oxygen species (ROS) production increased in *Slamf1*^*-/-*^ compared to BALB/c in 5 out of 6 strains. However, *Slamf1*^*-/-*^macrophages infected with VFRA strain exhibited a divergent behavior, with higher parasite load, lower NOX2 expression and ROS production compared to BALB/c. Parasitological and immunological studies *in vivo* with Y strain showed that in the absence of SLAMF1 the immune response protected mice from the otherwise lethal Y infection favoring a proinflammatory response likely involving CD4, CD8, dendritic cells and classically activated macrophages. In the case of VFRA, no major changes were observed in the absence of SLAMF1. Thus, the results suggest that the *T*. *cruzi* affects SLAMF1-dependent ROS production, controlling parasite replication in macrophages and affecting survival in mice in a strain-dependent manner. Further studies will focus in the identification of parasite molecules involved in SLAMF1 interaction to explain the immunopathogenesis of the disease.

## Author summary

Chagas disease, caused by *Trypanosoma cruzi*, is characterized by an acute phase, with low mortality, and after many years without any sign of disease, patients develop a symptomatic chronic phase, characterized by cardiomyopathy and/or digestive mega syndromes. These differences have been attributed to the high genetic variability of this parasite. We have shown that the receptor Signaling Lymphocyte-Activation Molecule Family 1 (SLAMF1) controls susceptibility to Infection by the lethal *T*. *cruzi* Y strain. Here we studied in detail the immunopathogenic role of SLAMF1 using 6 genetically diverse strains of *T*. *cruzi* using *in vitro* and *in vivo* approaches. Our results indicate an important role of SLAMF1 in *T*. *cruzi* infection which is parasite strain-dependent. We found that parasites interact with SLAMF1 in macrophages affecting NADPH oxidase (NOX2) expression and reactive oxygen species (ROS) production 5 out of 6 strains tested. Y and VFRA strains showed a divergent behavior *in vitro* and the role of SLAMF1 in the *in vivo* infection was also strikingly different. The Y strain caused 70% mortality in BALB/c mice but not in *Slamf1*^*-/-*^ mice. The proinflammatory response was stronger in the last, suggesting that SLAMF1 was repressing protective immune responses of mice infected with the Y strain. In contrast, for VFRA, SLAMF1 deficiency resulted in 100% survival of BALB/c mice, without major changes in the immune response in the absence of SLAMF1. Thus, the results indicate that SLAMF1 receptor interacts with *T*. *cruzi*, affecting parasite replication and ROS production in macrophages as well as the adaptive immune response in mice in a parasite strain-dependent manner. Future studies will focus in understanding the immunopathogenic role of SLAMF1 during *T*. *cruzi* infection.

## Introduction

Chagas disease is an important problem of public health caused by the intracellular parasite *Trypanosoma cruzi*. It is a complex disease with a great variety of reservoirs and vectors and different ways of transmission. Clinically there are 2 main phases, the acute phase characterized by local inflammation and fever, and chronic which can be asymptomatic or with cardiac, digestive and/or cardiodigestive clinical symptoms [1]. Parasite isolates show high genetic variability and at least 6 different Discrete Units (DTUs) have been established. A relation between DTUs and clinical symptoms has been hypothesized [2]. Despite experimental infections in mice with different parasite strains produce different symptoms underlying different pathogenic mechanisms [3], results in humans do not clearly support this hypothesis [4]. Thus, the immunopathology is still not completely understood.

We previously reported that Signaling Lymphocytic Activation Molecule 1 (SLAMF1 or CD150), a receptor expressed almost exclusively in hematopoietic cells [5], controls susceptibility to *T*. *cruzi* infection with the virulent Y strain [6]. The SLAM family of receptors is composed of 9 members with a similar structure. SLAMF1-7 genes are located in the same cluster in chromosome 1 and contain intracellular ITAM motifs. But SLAMF8-9, nearby both in human and mouse, lack intracellular Immuno-receptor tyrosine-based activation motif (ITAM). Interaction of OmpC/F+*E*. *coli* with SLAMF1 is required for macrophage phagocytosis and phagosome localization where it enhances PI3P production, activation of NADPH oxidase (NOX2) and superoxide production. NOX2 is a multi-subunit enzyme with membrane and cytosolic components, which actively communicate during the host responses to a wide variety of stimuli and mediates ROS production, which has an important role in the elimination of invading microorganisms in macrophages and neutrophils [7]. Superoxide produced by the NOX2 complex, besides antimicrobial activity, is a signaling molecule that modulates cell motility and phagocytosis, thus, in the absence of SLAMF1 the phagocytic process of Gram- bacteria is compromised [8]. SLAM-family receptors expressed by different cells can form homophilic interactions between them, recruiting adaptor proteins as SLAM-associated adaptor (SAP) and Fyn in T cells that enhance IL4 and IL13 production [9]. However, a different adaptor, EAT-2 is involved in SLAMF1 signaling in natural killer (NKs) and antigen-presenting cells (APCs) [8]. During inflammation, phagocytes initially increase SLAMF1 surface expression and cell motility and subsequently induce SLAMF8 expression with a negative effect on cell motility, constituting a rheostat-like mechanism that modulates inflammation [10]. SLAM receptors are direct microbial sensors that can benefit pathogens allowing infection, as for measles virus [11], or the host, as in bacterial infections [12]. This is likely due to different interacting molecules since the measles virus has SLAMF-like structures that favor their entrance into cells competing with endogenous SLAMF1 receptors, modulating their function.

Here we investigated the interaction of SLAMF1 receptor with *T*. *cruzi* and the parasitological and immunological effects of SLAMF1 deficiency *in vitro* and *in vivo*. In addition, given that *T*. *cruzi* isolated strains present great genetic variability and cause very different infection outcomes [13], we aimed to investigate whether the host-detrimental role of SLAMF1 observed with the Y strain, can be extended to other *T*. *cruzi* strains. First, we studied the behavior *in vitro* of six parasite strains representative of each DTU (Dm28c, Y, M6421 cl6, 10R26, Bug2148 cl1 and VFRA cl1) in macrophages isolated from BALB/c and *Slamf1*^*-/-*^ mice. We found two major types of macrophage responses associated to infection with different parasite strains. Finally, representative parasite strains with divergent behavior were selected for their study in experimental mouse infections.

## Materials and methods

### Ethics statement

This study was carried out in strict accordance with the European Commission legislation for the protection of animal used purposes (2010/63/EU). The protocol for the treatment of the animals was approved by the *Comité de Ética de la Dirección General del Medio Ambiente de la Comunidad de Madrid*, Spain (permits PROEX 21/14 and PROEX 148/15). Animals had unlimited access to food and water. They were euthanized in a CO_2_ chamber and all efforts were made to minimize their suffering.

#### Parasites

The Y strain was obtained from Dr. J. David (Harvard Medical School, Boston, Massachusetts, USA), originally isolated back in 1953 [14]. Dm28c, M6421 cl6, 10R26, Bug2148 cl1 and VFRA cl1 strains were obtained from Dr. M. Miles (London School of Hygiene and Tropical Medicine, London, UK) through the European program ChagasEpiNet. Discrete Typing Unit (DTU) classification, abbreviations, geographic origin, and biologic origin are shown in Table 1.

**Table 1:** *T. cruzi* strains. Discrete Typing Unit (DTU) classification, abbreviation used in the manuscript, geographical and biological origins are shown for each of the *T. cruzi* parasite strains.

DTU	Strain	Abbreviation	Geographical origin	Biological origin
TcI	Dm28c	Dm28	Carabobo, Venezuela	*Didelphis marsupialis*
TcII	Y	Y	Sao Paulo, Brazil	*Homo sapiens*
TcIII	M6421 cl6	M6421	Belem, Brazil	*Homo sapiens*
TcIV	10R26	10R26	Santa cruz, Bolivia	*Aotus sp*.
TcV	Bug2148 cl1	Bug	Rio Grande do Sul, Brazil	*Triatoma infestans*
TcVI	VFRA cl1	VFRA	Francia, Chile	*Triatoma infestans*

#### Mice

BALB/c were purchased from Charles River Laboratories and *Slamf1*^*-/-*^ mice were kindly provided by Dr. Terhorst (USA), all were maintained under pathogen-free conditions at *Centro de Biología Molecular Severo Ochoa* (CSIC-UAM) animal facility.

### Vero cells/ trypomastigote co-cultures

Vero cells (ATCC number CCL-81) were grown in 100 mm tissue culture plates with complete Roswell Park Memorial Institute (RPMI, Thermo Fisher Scientific) medium containing 2 mM L-glutamine, 100 U/ml penicillin, 100 μg/ml streptomycin and 0.1 mM non-essential amino acids and supplemented with 5% Fetal Bovine Serum (FBS) at 37°C in the biosafety level 3 (BSL3) cell culture laboratory at *Centro de Biología Molecular Severo Ochoa* (CSIC-UAM). *T*. *cruzi* trypomastigotes of all the strains obtained from frozen supernatants of previous infections of Vero cells were used to re-infect them. After 2–3 days, *T*. *cruzi* trypomastigotes released into the supernatants were collected, pelleted by centrifugation (at 720 g, for 10 min at room temperature), resuspended in complete RPMI medium and used for infection of macrophages. When indicated, trypomastigotes of the Y strain obtained from Vero cells were labelled with fluorescent probes Mito Tracker Orange (Thermo Fisher Scientific) or Mito Tracker Deep Red (Thermo Fisher Scientific), at a concentration of 1 μM for 1 h, pelleted by centrifugation and then washed with Phosphate-Buffered Saline (PBS) twice to remove the free probe.

### Thioglycollate-elicited peritoneal macrophage infection

BALB/c and *Slamf1*^*-/-*^ 8–12 weeks old female mice were injected intraperitoneal with 10% thioglycollate (Gibco, Grand Island, NY) (1 ml per mouse). After 4 days, mice were euthanized by CO_2_ inhalation. Peritoneal cells collected by lavage with 10 ml of cold PBS were pelleted by centrifugation (260 g, for 10 min, at 4°C) and seeded in 6 well multi-well plates (10^6^ cells/well) in RPMI medium supplement with 5% FBS overnight at 37°C. Non-adherent cells were removed by gently washing three times with warm PBS. Peritoneal macrophages were infected with trypomastigotes of *T*. *cruzi* at a ratio of 5 parasites per cell. At 1, 6 and 24 hours post-infection (hpi), the cells were washed three times with PBS to remove the unbound parasite.

### Fluorescence microscopy

Thyoglicollate-elicited macrophages from BALB/c and *Slamf1*^*-/-*^ mice were cultured overnight in 24-well multi-well plates (2x10^5^ cells / well) containing 13 mm diameter coverslips in complete RPMI medium 5% FBS at 37°C and 5% CO_2_. Then, they were cultured with previously labelled trypomastigotes of the strain Y of *T*. *cruzi* with Mito Tracker Orange (Thermo Fisher Scientific) at ratio of 2 parasites per cell for 1 h. Coverslips were washed 3 times with PBS and the cells were fixed in 4% PBS paraformaldehyde (PFA) for 20 minutes at room temperature. The Dapi dye (1: 5,000; Calbiochem) was used to visualize the nuclei of the cells and parasites as well as the kinetoplast DNA. The samples were mounted on slides with Prolong Gold Antifade mounting medium (Invitrogen) and were observed and analyzed in the Axiovert200 inverted microscope (Zeiss), 630x magnification.

### SLAMF1-GFP expression vector

The human *SLAMF1* cDNA (Origene) was cloned into the commercial vector pcDNA3 (Invitrogen), using the enzymes NheI and Xmal. Similarly, the *GFP* cDNA was subcloned into the 3 'region of the aforementioned *SLAMF1* cDNA, to generate the SLAMF1-GFP fusion protein vector using the restriction enzymes Xmal and EcoRV.

### Cell transfection

HEK293T cells were grown in Dulbecco’s Modified Eagle Medium (DMEM, Thermo Fisher Scientific), containing 2 mM L-glutamine, 100 U/ml penicillin, 100 μg/ml streptomycin and 0.1 mM non-essential amino acids and supplemented with 5% FBS. SLAMF1-GFP vector transfection into HEK293T cells was performed using the Metafectene reagent (Biontex). Briefly, cells were seeded in 35mm / well of 6-well plates (2x10^5^ cells / well) and maintained in 5% FBS RPMI 1640 medium (GIBCO) without antibiotics overnight. Subsequently, the SLAMF1-GFP vector and Metafectene were separately diluted in RPMI medium without serum or antibiotics for 5 minutes at room temperature. Then the dilutedSLAMF1-GFP vector DNA (8μg) and Metafectene (20μl) were mixed for 20 minutes. After this time, the mixture was added to the cell culture and incubated at 37° and 5% CO_2_ for 24 h. Finally, the medium was replaced by complete RPMI medium 5% FBS and incubated 24 h at 37ºC 5%CO_2_.

### Flow Cytometry

5x10^5^ HEK293T cells transfected with SLAMF1-GFP plasmid (HEK SLAMF1 GFP) and HEK293T (HEK Control), were trypsinized at 48 hours after transfection and transferred from the plates to flow cytometer tubes, and were incubated for 1 hour with the different strains of *T*. *cruzi*, at a ratio of 5 parasites per cell, previously labelled with the fluorescent probe Mito Tracker Deep Red (Thermo Fisher Scientific). After the 1h incubation, the cells were washed twice (400g, 5 min at 22ºC) in order to eliminate the excess parasites not interacting with the cells, and fixed in 4% PFA in PBS for 20 minutes at room temperature. Subsequently, they were washed twice (400 g, 5 min at 22ºC) with PBS buffer and resuspended in PBS. The samples were analyzed on the FACSCalibur flow cytometer (Becton Dickinson) and the data was analyzed with the FlowJo 7.0 program (Tree Star, Inc. Oregon Corporation).

### DNA purification

DNA was obtained using the High Pure Template preparation kit (Roche Life Science) following the directions of the manufacturer for DNA purification. DNA was purified from macrophages seeded in 60 mm tissue culture plates that were scrapped and from 25–35 mg of spleen, liver, intestine and heart and the DNA was quantified in a Nanodrop (Thermo Scientific) and kept at -80°C.

### Parasite load determination by quantitative real-time PCR (qPCR)

qPCR reactions were conducted with DNA from macrophages, spleen, liver, intestine and heart tissues (100 ng) using a modification of the method described by Piron *et al*., 2007 [15]. Briefly, GoTaq qPCR Master Mix (Promega) was used in qPCR reactions with oligonucleotides derived from satellite DNA were used (sense: 5’-ASTCGGCTGATCGTTTTCGA-3’; and anti-sense: 5’-AATTCCTCCAAGCAGCGGATA-3’; were S is C/G). As a loading control oligonucleotides derived from mouse Tumor Necrosis Factor (*Tnf*) were used (sense: 5’-CCCTCTCATCAGTTCTATGGCCCA-3′; and anti-sense: 5’-CAGCAAGCATCTATGC ACTTAGACCCC-3’). Parasite load was calculated from the comparative threshold cycle (C_T_) values obtained from *T*. *cruzi* primers and normalized with respect mouse *Tnf* C_T_ values as described [16]. The regression equation resulted from plotting the C_T_ values obtained from the standard curve of 10-fold serial dilutions starting from 1,000 pg to 0.001 pg of parasite DNA. Results were expressed as pg of *T*. *cruzi* DNA per ng of total DNA.

### RNA purification

RNA purification was performed using the TRIzol reagent (Invitrogen) following manufacturer instructions. Non-infected macrophages and those infected with the different *T*. *cruzi* strains (Dm28, Y, M6421, 10R26, Bug2148 and VFRA) as well as HEK Control and HEK SLAMF1 GFP cells were scrapped from 60 mm tissue culture plates and transferred to tubes. 25–35 mg of tissues as heart, gut, spleen and liver tissues were cut using a sterile scalpel blade and disrupted using a PT 1300D homogenizer (Kinematica Polytron, Fisher Scientific) as previously described [17]. The purified RNA obtained was quantified in the Nanodrop (Thermo Scientific) and kept at -80°C.

### Gene expression determination by Quantitative Reverse–Transcription (RT-qPCR)

For RT-qPCR analysis, reverse transcription of total RNA was performed using the High Capacity cDNA Archive Kit (Applied Biosystems) and the amplification of different enzymes and cytokines genes was performed using gene-specific oligonucleotide (S1 Table) in triplicate reactions with GoTaq qPCR Master Mix (Promega) on an ABI PRISM 7900 HT instrument (Applied Biosystem, Life Sciences). The relative quantity of each gene expression was calculated by the comparative threshold cycle (C_T_) method following the protocol instructions. All quantifications were normalized to the ribosomal mouse *18s* or human *18S* gene, as indicated, to account for the variability in the initial concentration of RNA and in the conversion efficiency of the reverse transcription (ΔC_T_). Finally, all data from samples taken from infected macrophages or infected mice were normalized respect to the values obtained from non-infected macrophages or non-infected mice (ΔΔC_T_). The relative quantity (RQ) was calculated as RQ = 2^-ΔΔCt^ as previously described [17].

### Reactive oxygen species assay

Peritoneal macrophages BALB/c and *Slamf1*^*-/-*^ were infected with trypomastigotes of the *T*. *cruzi* strains Dm28, Y, M6421, 10R26, Bug2148 and VFRA in a rate of 5 parasites per cell. At 6 hpi the cells were washed with PBS three times to remove unbound parasite and RPMI medium without Phenol Red (RPMI-PhR) was added. Macrophages were detached by gently tapping the plate and pipetting the medium over the cell monolayer from the 6 well plate with 1 ml of RPMI-PhR and transferred to cytometry tubes. 2 μl of CellROX Green Reagent (Thermo Fisher Scientific) was added to the samples to a final concentration of 250 μM and incubated for 30 min at 37°C. The medium was discarded and the samples were collected, pelleted by centrifugation (720 g, 10 min. 37°C) and washed three times with PBS 1X following by centrifugation (720 g, 10 min. 37°C). The samples were fixed with paraformaldehyde 1% in PBS 20 min at 4°C protected from light. Finally, the samples were analyzed by flow cytometry in the FACSCanto II (BD Biosciences) and the data were analyzed with the FlowJo software (Tree Star, Inc. Oregon Corporation).

### Blood *T*. *cruzi* trypomastigotes

To obtain blood of *T*. *cruzi* trypomastigotes of the Y and VFRA strains for inoculating experimental groups of mice, we maintained them by periodic passages of at least 2x10^3^ trypomastigotes in 2 BALB/c mice via intraperitoneal. To increase the number of parasites obtained from blood, infected mice were injected intraperitoneal with a single dose (200 mg/kg of body weight) of cyclophosphamide (Sigma Aldrich) and parasites were elicited after 5 days as described [18].

### Experimental infections in mice

*In vivo* experiments were performed in Biosafety level 3 (BSL3) animal facility at *Centro de Biología Molecular Severo Ochoa* (CSIC-UAM). Groups of BALB/c and *Slamf1*^*-/-*^ mice were injected intraperitoneally with 2x10^3^ blood trypomastigotes per mouse with Dm28, Y and VFRA strains, in two independent experiments using 3 mice for each condition; one mice of each group was processed for Hematoxylin & Eosin staining of heart tissue (H&E, Sigma-Aldrich), consequently many parameters were determined in experimental groups of 5 mice (n = 5), as indicated. Mice were euthanized at the early acute phase (EA) at 14 days post-infection (dpi) and the late acute phase (LA) (16–21 dpi), with their respective control non-infected mice, by CO_2_ inhalation. Mice and organs (spleen, liver, and heart) were weighted and intestines were elicited. All organs were frozen at -80ºC.

### Statistical analysis

Results from macrophage infections are representative of at least two experiments performed in triplicate (n = 6). Two experiments were performed *in vivo*. Data from groups of 3 mice from one experiment and groups of 2 mice from another experiment (n = 5) were analyzed and data represented in bar graphs as mean ± Standard Error of the mean (SEM) or in box plots.

Statistical significance was evaluated by student´s two-tailed *t-*test (95% confidence interval) with the GraphPad Prism version 5.0 for Windows (GraphPad Software, San Diego California USA). Heat maps and Principal Components Analysis (PCA) analysis of gene expression in macrophages, intestine and heart tissues were performed using R and Rstudio. The strain names are located in the centroid of the cluster of individual mice data in the PCA graphs. For each variable analyzed we considered contributions above 10% of the variance in either dimension 1 (PC1) or dimension 2 (PC2) to be significant.

## Results

### Interaction of *T*. *cruzi* with SLAMF1

**Fig 1:**
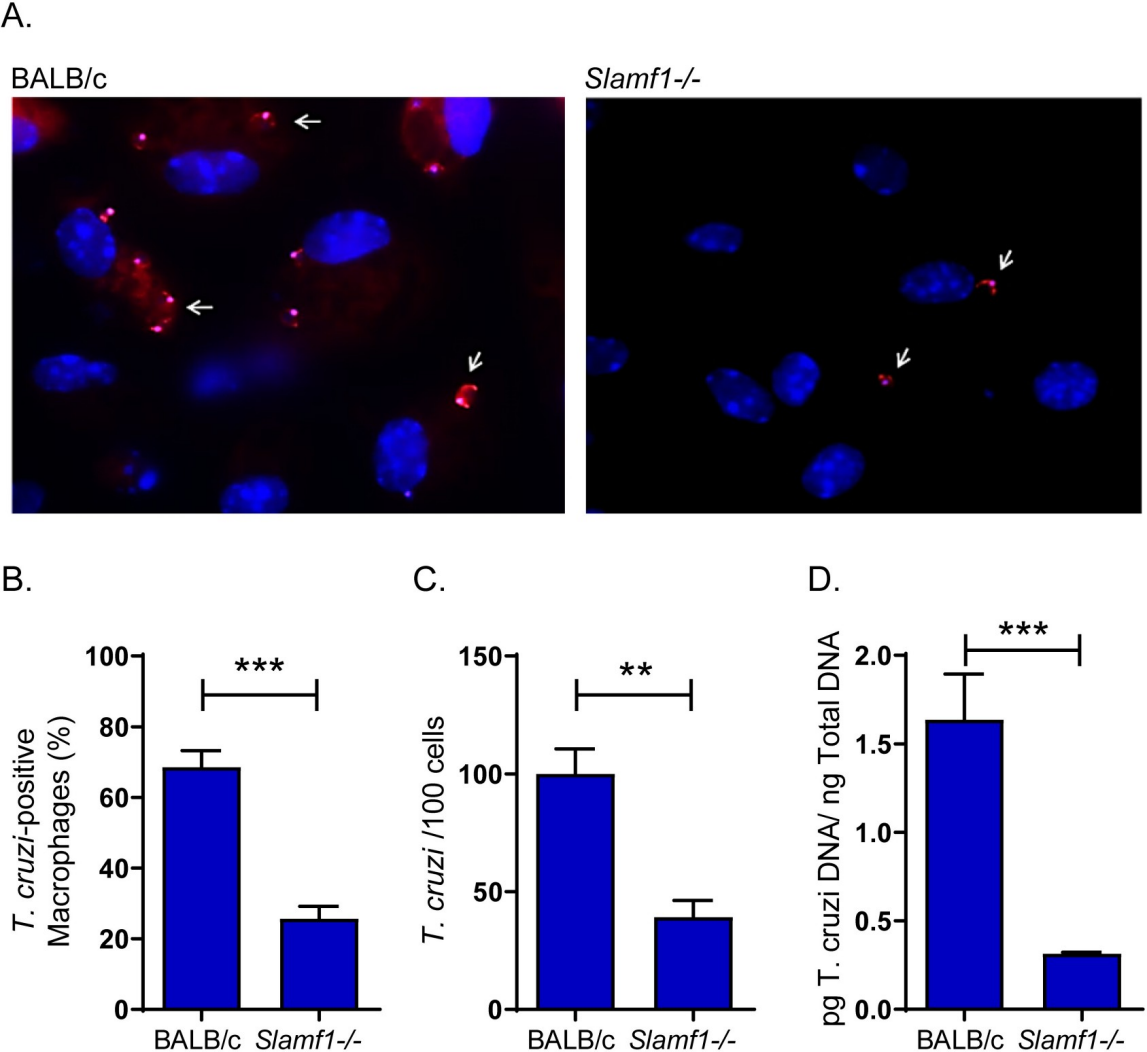
Interaction of *T. cruzi* with macrophages from BALB/c and Slamf1^-/-^ mice. A) Fluorescence microscopy images corresponding to BALB/c and Slamf1^-/-^ macrophages incubated for 1 h with parasites of the Y strain. The nucleus of macrophages appears stained in blue (Dapi). The arrows point to fluorescent parasites labelled with Mito Tracker Orange attached to macrophages from BALB/c and Slamf1^-/-^ mice. A representative experiment of two is shown. B) Percentage of peritoneal macrophages from BALB/c and Slamf1^-/-^ mice interacting with *T. cruzi* after 1 h. C) Number of trypomastigotes attached to 100 peritoneal macrophages from BALB/c and Slamf1^-/-^mice after 1 h. In B and C the mean ± SEM corresponds to 14 fields of each condition performed in duplicate. D) Quantification of the parasite load in macrophages from BALB/c and Slamf1^-/-^ mice incubated with the Y strain during 1h. The statistical significance of the differences between both strains of mice is indicated: *** p <0.001, ** p <0.01.

To study the interaction of SLAMF1 with the parasite, peritoneal macrophages from BALB/c and *Slamf1*^*-/-*^ mice were incubated with trypomastigotes labelled with a fluorescent reagent (Fig 1A). Quantification of the macrophage-parasite direct interaction through the images obtained revealed that 68% of BALB/c macrophages were *T*. *cruzi*-positive, compared to 25% of *Slamf1*^*-/-*^ (Fig 1B). Furthermore, around 100 *T*. *cruzi* trypomastigotes were detected in 100 BALB/c infected macrophages but the number decreased to around 38 trypomastigotes in 100 *Slamf1*^*-/-*^ macrophages (Fig 1C). In addition, parasite binding was determined by qPCR, showing a 5-fold decrease in the absence of SLAMF1 (Fig 1D).

**Fig 2:**
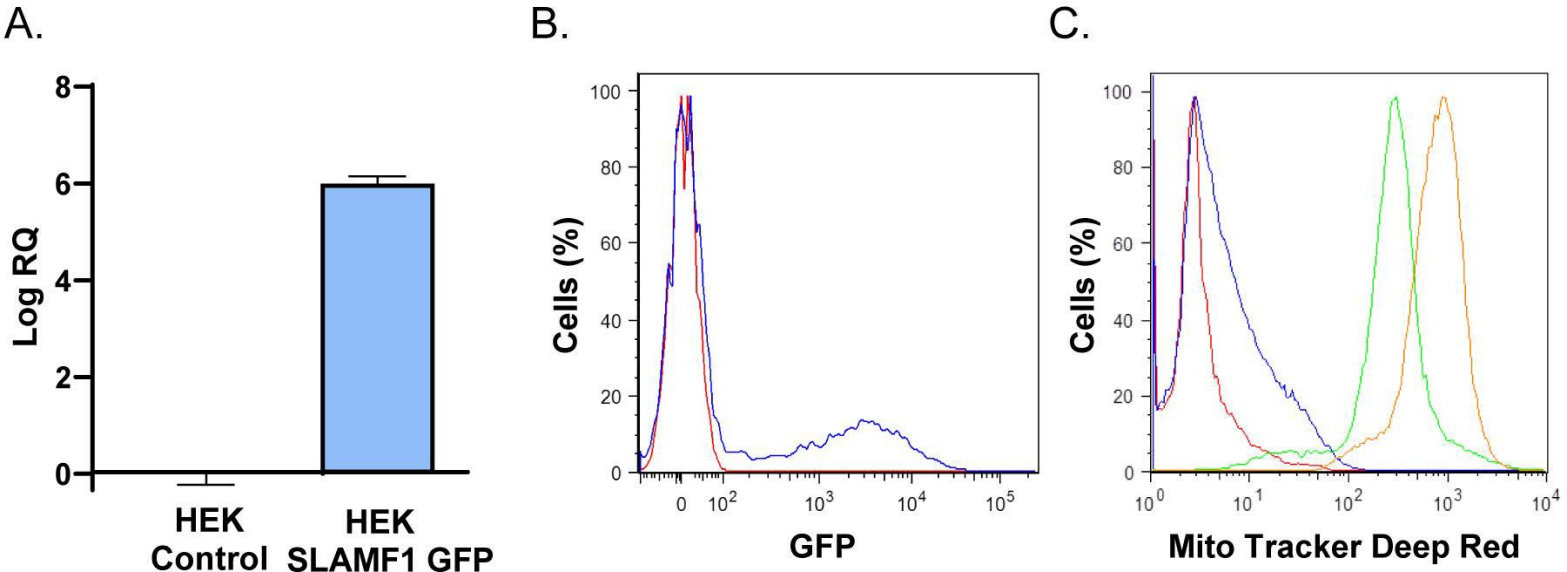
Interaction of *T. cruzi* with HEK293T cells over-expressing SLAMF1-GFP. A) Quantification by RTqPCR of the relative expression of SLAMF1-GFP mRNA respect to human 18S mRNA in HEK Control and HEK SLAMF1 GFP cells. B) Flow cytometry analysis of SLAMF1 Protein expression in HEK Control cells (Red line) and HEK SLAMF1 GFP cells (blue line). C) Flow cytometry analysis of HEK Control cells (green line) and HEK SLAMF1 GFP cells (orange line) incubated with *T. cruzi* trypomastigotes labelled with Mito Tracker Deep Red); HEK Control cells (Red line) and HEK SLAMF1 GFP cells (blue line) were not incubated with labelled parasites. A representative result out of two independent experiments is represented.

To confirm the previous results, a parasite binding assay was set up using parasites previously labelled with a fluorescent reagent and HEK293T (HEK) cells transfected with SLAMF1-GFP expression vector. The results by RTqPCR (Fig 2A) and flow cytometry (Fig 2B) analysis showed the overexpression of SLAMF1 GFP. Higher fluorescence intensity due to parasite interaction was detected in HEK293T SLAMF1 GFP compared to HEK293T Control cells (Fig 2C)

### The macrophage response to infection of depends on the *T*. *cruzi* parasite strain and SLAMF1 expression

**Fig 3:**
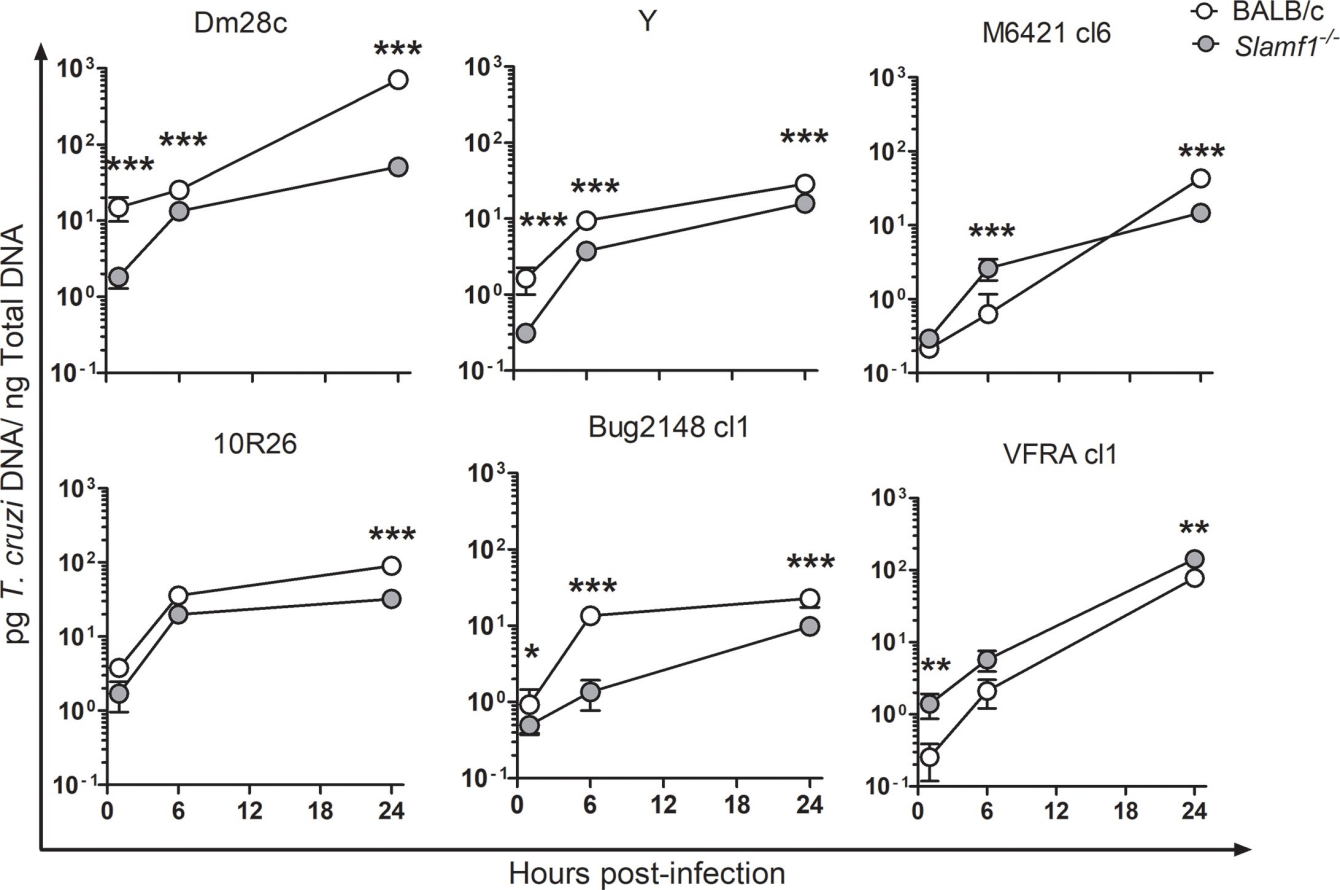
Parasite load in Slamf1^-/-^ and BALB/c peritoneal macrophages. Analysis of parasite load of BALB/c and Slamf1^-/-^ peritoneal macrophages at 1, 6 and 24 hpi with different parasite strains. Parasite load was quantified by qPCR. Mean and Standard error of the mean ± (SEM) of two independent experiments in triplicate is represented (n = 6). The asterisks indicate the statistical significance, t student (*p<0.05, **p<0.005 and ***p<0.001), between BALB/c and Slamf1^-/-^ macrophages.

We infected BALB/c and *Slamf1*^*-/-*^ peritoneal macrophages with 6 different parasite strains and intracellular parasite load was determined at 1, 6 and 24 hpi (measured as pg of *T*. *cruzi* DNA per ng total DNA). BALB/c macrophages presented great strain-dependent variations in parasite load at all-time points analyzed, in particular at 24 hpi it was highest for Dm28c (709 pg/ng) and lowest for Bug2148 cl1 (22 pg/ng) (Fig 3). At 1 and 6 hpi *Slamf1*^*-/-*^ macrophages infected with 3 strains (Dm28, Y and Bug2148) showed a significant reduction in parasite load respect to BALB/c (Fig 3). On the contrary, VFRA and M6421, showed higher parasite loads in *Slamf1*^*-/-*^ macrophages than in BALB/c at 1 and 6 hpi. Finally, at 24 hpi *Slamf1*^*-/-*^ macrophages infected with all parasite strains showed lower loads respect to BALB/c macrophages, except VFRA that showed an opposite behavior. Notably, lower parasite loads were observed in *Slamf1*^*-/-*^ macrophages at 1 hpi, in all the strains except VFRA for which it was higher.

### Analysis of the immune status in infected macrophages

Next, we analyzed the mRNA expression of key molecules involved in macrophage activation or related with SLAMF1 signaling, as Arginase 1 (*Arg1*), NADPH oxidase 2 (NOX2, *Cybb* subunit), Immuno-responsive gene 1 (*Irg1*) and cytokines as Interleukin (IL)1β (*Il1b*), IL6 (*Il6*), IL10 (*Il10*) and Tumor necrosis factor (*Tnf*) (S1 Fig).

**Fig 4:**
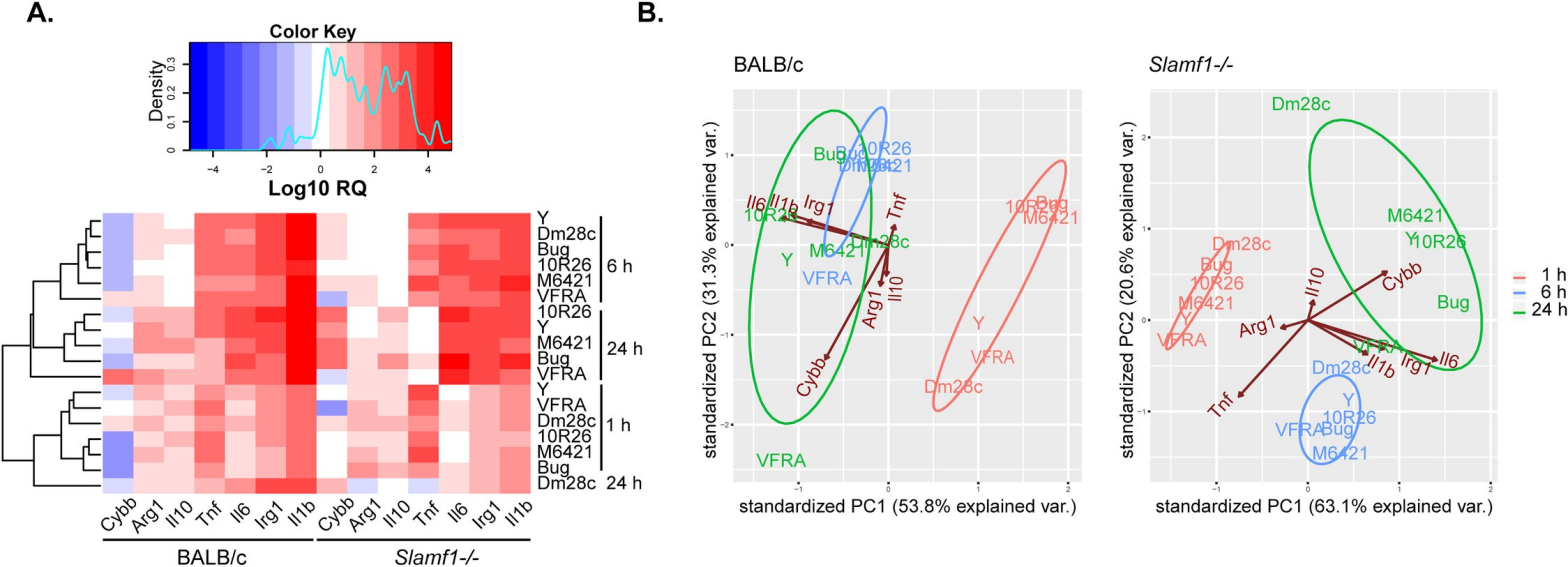
Gene expression in BALB/c and Slamf1^-/-^ peritoneal macrophages infected with different strains of *T. cruzi*. A) Heat map of gene expression was determined by RTqPCR and RQ values were obtained after normalizing values from infected respect to non-infected macrophages. Results of two independent experiments in triplicate are represented (n = 6). For each gene, a color code was given from high (Red) to low (Blue). B) PCA analysis was performed for BALB/c and Slamf1^-/-^ macrophages.

A heat map of gene expression of BALB/c and *Slamf1*^*-/-*^ macrophages infected with the different strains of *T*. *cruzi* is shown and indicates common as well as different patterns between them (Fig 4A). Data from different parasite strains clustered according to different hpi in BALB/c and *Slamf1*^*-/-*^ macrophages, except for Dm28 infected macrophages at 24 hpi. Notably, the basal gene expression of *Slamf1*^*-/*-^ respect BALB/c non-infected macrophages was similar for *Irg1*, slightly lower for *Arg1*, *Il1b*, *Il6*, and *Il10*, but higher for *Cybb* and *Tnf* (S2 Fig).

To emphasize variations and bring out strong patterns in our gene expression data, we performed PCA analysis (Fig 4B and S2 Table). PC components explained 85.1% and 83.7% of the variance in BALB/c and *Slamf1*^*-/-*^ macrophages, respectively. We found that at 1 and 6 hpi all parasite strains grouped according to the time post-infection in BALB/c and *Slamf1*^*-/-*^ macrophages, but principal changes were found in BALB/c at 24 hpi. At this time, the pattern observed in BALB/c macrophages was defined by *Il1b* / *Il6* / *Irg1*, with all the strains, except VFRA which exhibited a pattern defined only by *Cybb*. In consequence, VFRA was not grouped with the rest of strains.

On the other hand, *Slamf1*^*-/-*^ PCA analysis corroborated heat map expression findings. From now on and throughout the manuscript, the gene expression patterns observed in *Slamf1*^*-/-*^ after PCA analyses correspond, unless indicated, to increases in gene expression respect to BALB/c. According to this, a pattern defined by *Tnf* was observed for all the strains at 1 and 6 hpi. But at 24 hpi *Cybb* pattern was observed for Y, M 6421, 10R26 and Bug2148 infecting strains but not for Dm28c and VFRA, which centroids were very distant in the PCA analysis respect to other strains. Moreover, *Il6* / *Irg1* pattern was observed for VFRA at 24 hpi. These results prompted us to further investigate the role of NOX2 in infected macrophages.

### SLAMF1 effect on ROS production is *T*. *cruzi* strain-dependent

**Fig 5:**
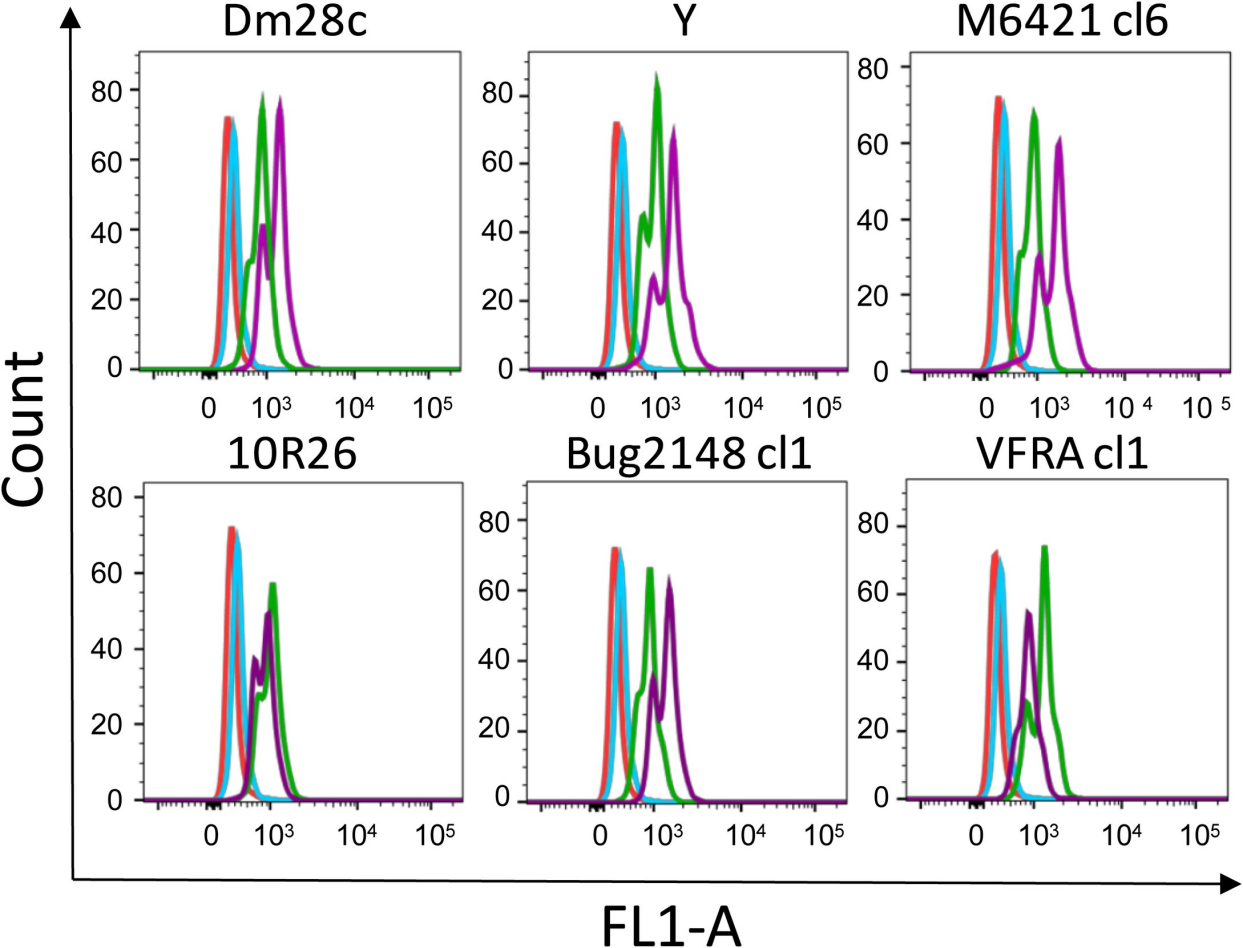
ROS production in macrophages at 6 hpi. Flow cytometry Assay for ROS detection in Slamf1^-/-^ and BALB/c peritoneal macrophages infected with different parasite strains. The diagrams show non-infected BALB/c macrophages (Red line), non-infected Slamf1^-/-^ (Blue line), BALB/c (Green line) and Slamf1^-/-^ (Purple line) infected with the different strains of *T. cruzi*.

We infected BALB/c and *Slamf1*^*-/-*^ macrophages with the 6 parasite strains and measured ROS production at 6 hpi by flow cytometry. ROS production in *Slamf1*^*-/-*^ macrophages infected with Dm28, Y, M6421 and Bug2148 strains increased respect to BALB/c, did not change with 10R26 and it was indeed reduced with VFRA, indicating a strain-dependent negative association between parasite load, *Cybb* expression and ROS production that depends on SLAMF1 expression (Fig 5).

### Role of SLAMF1 in *T*. *cruzi* replication *in vivo*

**Fig 6:**
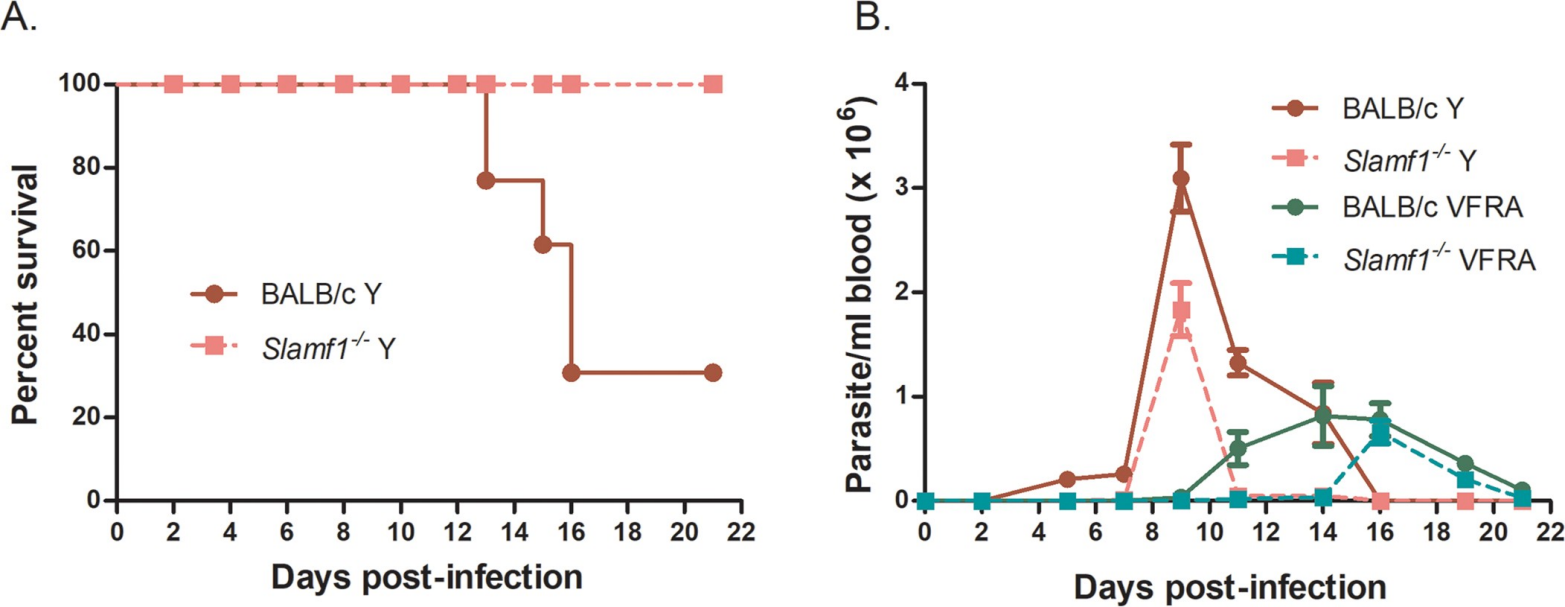
Survival and parasitemia of BALB/c and Slamf1^-/-^ mice infected with Y and VFRA strains of *T. cruzi*. A) Survival was monitored from 0 to 21 dpi. Only the Y strain caused the death of BALB/c mice. B) Parasitemia was monitored every 2 or 3 days; Mean parasitemia of 5 mice per group infected with Y and VFRA strains is shown.

In order to study the role of SLAMF1 in experimental infections, we selected the two strains, Y and VFRA, that had the most striking different behavior in macrophages regarding to parasite load and their effect on macrophage NOX2 expression and ROS production. Besides, Y and VFRA result in detectable parasitemia [3, 13]. Moreover, they also have different behavior in *in vivo* infection in BALB/c mice, being the Y strain lethal [6] and VFRA strain less virulent [13]. In agreement with that, 70% of BALB/c mice succumbed to infection with the Y strain [19], while 100% of *Slamf1*^*-/-*^ mice survived [6] (Fig 6A). In contrast, infection with VFRA strains neither caused mortality in BALB/c, nor in *Slamf1*^*-/-*^ mice. Notably, *Slamf1*^*-/-*^ mice showed lower parasitemia than BALB/c mice with both parasite strains. BALB/c and *Slamf1*^*-/-*^ mice infected with the Y strain presented the first peak of parasitemia at 9 dpi, and with VFRA it was delayed to 14–16 dpi (Fig 6B). In addition, since Dm28 was the strain presenting the highest parasite load in infected macrophages, we also infected the mice with this strain. But in this case, all mice survived and no parasitemia was detected (not shown).

Splenomegaly was observed in infected mice (S3A Fig) and it was higher in BALB/c mice than in *Slamf1*^*-/-*^ mice, and higher with the Y strain compared to Dm28 and VFRA (S3A and S3B Fig). Parasite load was higher in BALB/c than in *Slamf1*^*-/-*^ spleens only with the Y strain at early acute (EA) phase. In *Slamf1*^*-/-*^ mice infected with the Y strain, we found lower parasite loads than with VFRA in the late acute (LA) phase and Dm28 showed the lowest parasite loads compared to Y and VFRA (S3C and S3D Fig).

Hepatomegaly was also induced by infection with the two parasite strains in both strains of mice and it was significantly higher in BALB/c compared to *Slamf1*^*-/-*^ in mice infected with Dm28 and VFRA (S4A and S4B Fig). Parasite load in livers infected with Dm28 and Y strains was higher in BALB/c than *Slamf1*^*-/-*^ mice. On the contrary, livers of mice infected with VFRA presented higher parasite load in *Slamf1*^*-/-*^ than in BALB/c mice (S4C and S4D Fig).

Parasite load in the intestine of *Slamf1*^*-/-*^ mice was significantly lower than in BALB/c mice after infection with the Y strain at EA (S5A Fig) and LA phases (S5B Fig). Notably, infection with VFRA resulted in detectable, but very low parasite load, both in BALB/c and *Slamf1*^*-/-*^ intestines.

Cardiac hypertrophy was only observed at the LA phase, where BALB/c and *Slamf1*^*-/-*^ mice infected with the Y strain showed higher cardiac hypertrophy compared to VFRA. Besides, although cardiac hypertrophy of *Slamf1*^*-/-*^ was lower compared to BALB/c mice infected with Y, and higher with VFRA, there were no statistically significant differences between the two mouse strains. Dm28 showed the lowest ratio but significantly higher in the absence of SLAMF1 at the EA phase (S6A and S6B Fig).

Parasite load in the heart was significantly higher in BALB/c compared to the *Slamf1*^*-/-*^ mice infected with the Y strain at EA and LA phases, but on the contrary, they were lower in mice infected with VFRA at EA phase (S6C and S6D Fig). In the case of Dm28, parasite load in the hearts was lower compared to Y and VFRA. Moreover, we found that heart hypertrophy was significantly associated with parasite load, where the higher parasite load resulted in an overall higher heart weight/body weight ratio. Cardiac amastigote nests and inflammatory infiltrates were visualized in heart sections from a single mouse of each group: non-infected and infected with Dm28, Y and VFRA strains (S7 Fig).

### Analysis of immune/inflammatory status of the infected intestine

We analyzed selected immune-related genes for Y and VFRA infection (S3 Table). For T cells we selected *Cd4* and *Cd8*, and markers of CD4 T cell subpopulations as *Ifng* (Interferon-γ) and *Tnf* for T helper (Th)1, *Il13* for Th2, *Foxp3* (forkhead box P3), *Il10* and *Tgfb* (transforming growth factor β) for Treg and *Il6* and *Il17* for Th17, although the last was not detected in any intestine sample (not shown). We also studied the expression of specific markers as *Irg1* for classically (M1) and *Cd206* for alternatively (M2) activated macrophages, *Il4r* for Monocytic Myeloid-derived suppressor cells (M-MDSCs), *S100a9* for granulocytic MDSC (G-MDSCs) and *Cd11c* for dendritic cells (DCs). In addition, we analyzed molecules, mostly enzymes, that can be expressed by different myeloid populations, indicative of the macrophage activation/polarization status, as *Arg1*, *Ptges* (Prostaglandin E Synthase) *and Cd68* (M2 and MDSCs), *Nos2* (inducible nitric oxide synthase, M1 and MDSCs) *and Cybb* (M1 macrophages, DCs and G-MDSCs).

**Fig 7:**
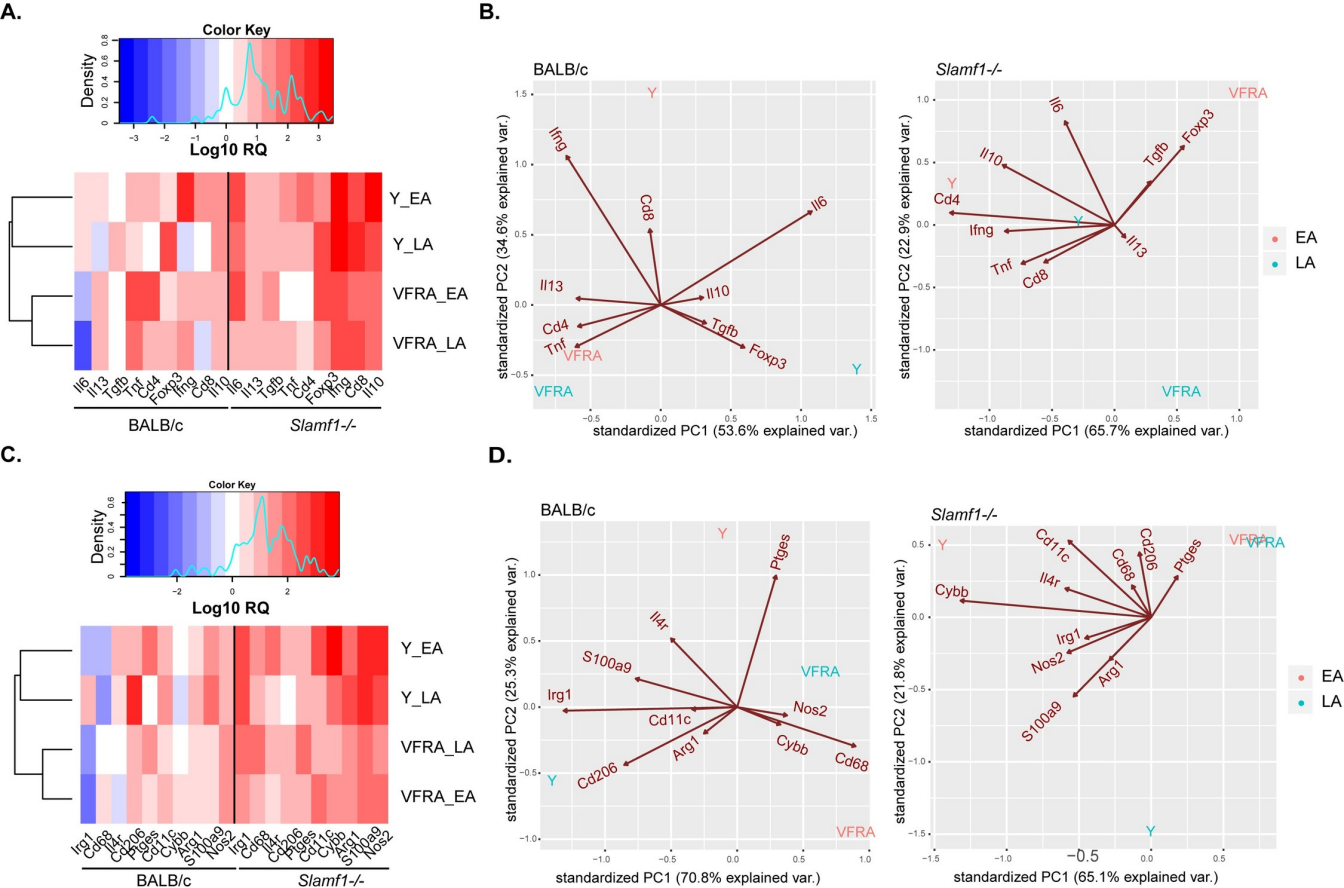
Gene expression in the intestine of BALB/c and Slamf1^-/-^ mice infected with Y and VFRA strains of *T. cruzi*. Gene expression was determined by RTqPCR and log RQ values were obtained after normalizing value from infected respect to non-infected intestines. Lymphoid population markers and cytokines heat map (A) and PCA analysis (B). Myeloid population markers and enzymes heat map (C) and PCA analysis (D). A color code was given from high (Red) to low (Blue) gene expression. Results corresponding to 5 mice per group are represented.

In order to detect variations, we represented separately the data of lymphoid markers from those of cytokines associated with T cells, which showed high fold changes (Fig 7A and 7B) and myeloid markers and enzymes with low fold changes (Fig 7C and 7D). Heat maps of gene expression allowed us to visualize gene expression results, and the alignment trees grouped the data according to the infecting strain (Fig 7A and 7C). For a complete interpretation of the results, we performed PCA analysis for lymphoid and myeloid markers. For lymphoid markers, the components explained 88.2% and 88.6% of the variance in BALB/c and *Slamf1*^*-/-*^ intestines, respectively. In BALB/c intestines infected with Y, *Ifng* / *Il6* showed greater contributions at the EA phase while *Foxp3* / *Il6* at the LA phase (Fig 7B and S4 Table). In the intestines of mice infected with VFRA, there was a contribution of *Cd4* / *Tnf* at EA and LA. In the absence of SLAMF1 the pattern with Y strain changed from *Ifng* / *Il6* observed in BALB/c intestines to *Ifng* / *Il6*/ *Il10* / *Cd4* / *Tnf*. In VFRA, the pattern changed from *Cd4* / *Tnf* to *Foxp3* (Treg) at the EA phase in the absence of SLAMF1.

For myeloid markers, PCA analysis explained 96.1% and 86.9% of the variance in BALB/c and *Slamf1*^*-/-*^ intestines, respectively (Fig 7D and S4 Table). We observed that in the EA phase of BALB/c mice infected with the Y strain the pattern was *Il4r* / *Ptges*, while in the LA phase *Cd206* / *Irg1*. But, in the absence of SLAMF1, gene expression pattern with Y shifted to *Cybb* / *Cd11c* in the EA phase and *Nos2* in the LA phase. There was no defined pattern in intestines infected with VFRA neither changes in the outcome of the disease.

### Analysis of immune/inflammatory status of infected hearts

**Fig 8:**
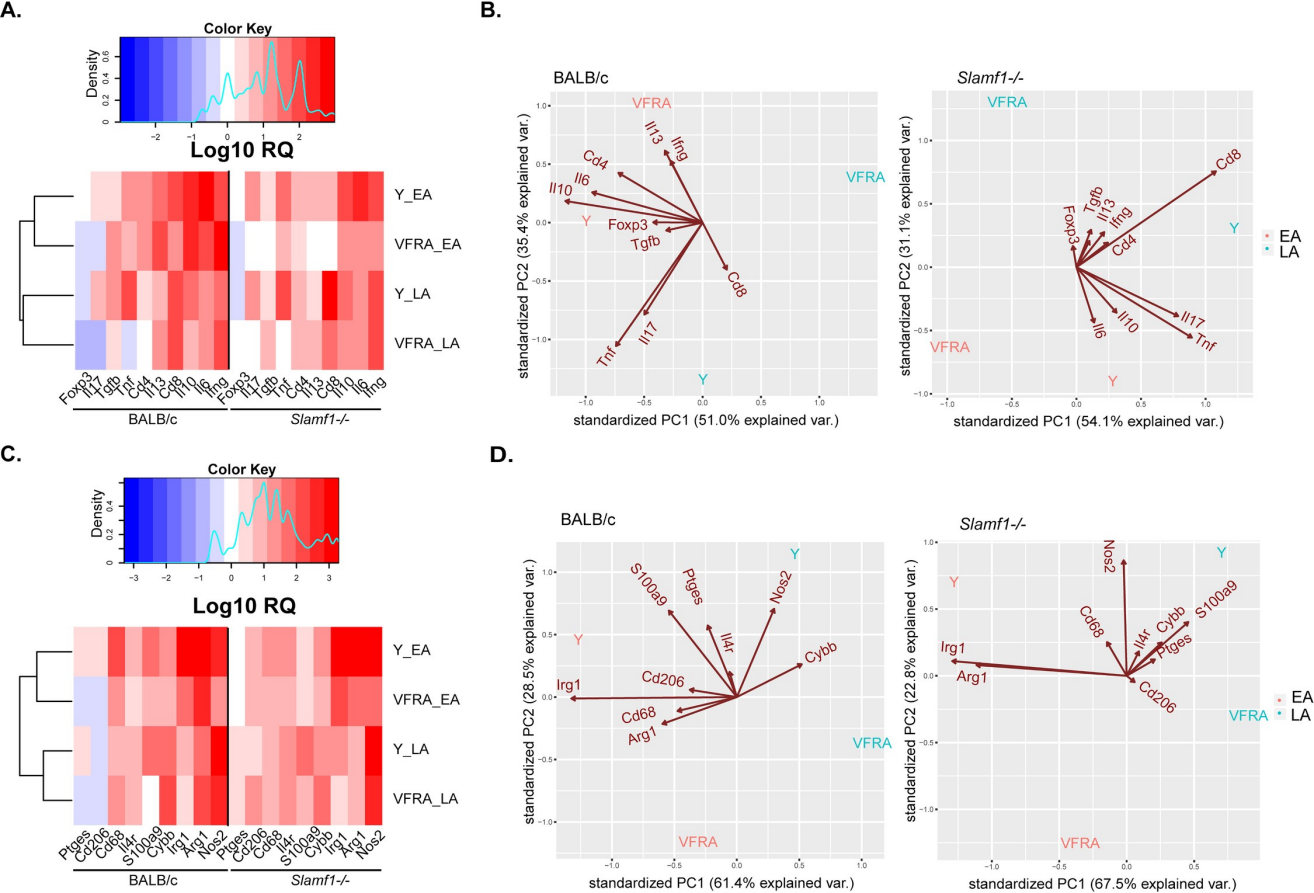
Gene heat map expression in heart of BALB/c and Slamf1^-/-^ mice infected with Y and VFRA strains of *T. cruzi*. Gene expression was determined in hearts of 5 mice BALB/c and Slamf1^-/-^ infected with Y and VFRA at the EA and LA phases by RTqPCR and RQ value was obtained after normalizing value from infected respect to non-infected hearts. Lymphoid population markers and cytokines (A) and PCA analysis (B). Myeloid population markers and enzymes (C) and PCA analysis (D). A color code was given in the heat maps from high (Red) to low (Blue) gene expression. Results corresponding to 5 mice per group are represented.

Heart gene expression for Y and VFRA was also performed (S5 Table). The heat map and the alignment trees for lymphoid markers showed an association between gene expression and phase of infection (Fig 8A). PCA analysis showed that the PCA components explained 86.4% and 85.2% of the variance in BALB/c and *Slamf1*^*-/-*^ hearts, respectively (Fig 8B and S6 Table). In heart tissue, the immune response was stronger in Y strain infected BALB/c mice characterized mostly by *Cd4* / *Il6* / *Il10* pattern in the EA phase, while there was an association with *Tnf / Il17* pattern at the LA phase. In contrast, in the EA phase the pattern induced by VFRA strain was defined by *Ifng*. No defined pattern was observed at the LA phase with VFRA.

Major changes were observed in *Slamf1*^*-/-*^ infected with the Y strain at the EA phase where the *Cd4* / *Il6* / *Il10* pattern observed in BALB/c was slightly decreased, while a small increase in *Cd8* expression was observed (Fig 8B and S6 Table).

For myeloid markers, the alignment tree also grouped the data according to the phase of infection (Fig 8C). PCA analysis showed that the PCA components explained 89.9% and 91.3% of the variance in BALB/c and *Slamf1*^*-/-*^ hearts, respectively (Fig 8D and S6 Table). In BALB/c hearts infected with Y in the EA phase, the pattern was *Irg1 / S100a9*, while at the LA phase it was *Ptges* / *Nos2*. No significant pattern was observed with VFRA. But in the absence of SLAMF1, in the EA phase in mice infected with Y there was an increase in *Irg1* with a decrease in *Arg1* expression, and in LA phase there was an increase of *Nos2* with a decrease in *S100a9* expression (Fig 8D and S6 Table). In addition, Arg*1*, *Irg1*, *Cybb*, *Il1b*, *Il6*, *Il10* and *Tnf* expression was analyzed in hearts of mice infected with Dm28, showing similar changes as the other strains but with less magnitude (S8 Fig).

## Discussion

The immunopathogenesis of Chagas disease is still not completely understood. A main reason is the high genetic variability of the parasite, with at least 6 different DTUs and many different isolated strains, that is hypothetically related to the different manifestations of the disease. Besides, in experimental infections in mice, different strains produce different symptoms and pathogenic mechanisms. Thus, identifying the molecules involved in pathogenesis and how they may relate with parasite genetic differences is important to further understand this complex disease. Previously, we found that SLAMF1 was a key in *T*. *cruzi* pathogenicity in the acute phase in mice [6]. Here we demonstrated a SLAMF1-dependent *T*. *cruzi* interaction by loss-/ gain-of-function using 3 different approaches. The results showed that there was more cell-parasite interaction when SLAMF1 was expressed.

Next, we addressed more in detail the immunopathogenic role of SLAMF1 using *in vitro* infection of macrophages with 6 genetically diverse parasite strains. The time point selection for *in vitro* experimental approach was based on the actual results where we detected parasites on the surface of cells up to 1 hpi and highly replicating intracellular parasites at 24 hpi [3]. Thus, we considered, 1h, as the time needed for full interaction, 1–6 hpi for internalization and 24 hpi for the first rounds of amastigote replication.

The results showed that different strains interact, internalize and replicate in BALB/c macrophages with varied efficiency, but we found decreased parasite loads in *Slamf1*^*-/-*^ macrophages infected with Dm28, Y, 10R26 and Bug2148 compared to BALB/c macrophages at 1 and 6 hpi, indicating that SLAMF1 is necessary for interaction and internalization in those strains. In contrast, M6421 and VFRA showed increased parasite loads, suggesting that in this case, SLAMF1 does not facilitate its interaction and internalization, rather the opposite. However, M6421 depended on SLAMF1 for replication, while with VFRA the parasite load was always enhanced in the absence of SLAMF1. These results suggest that SLAMF1 is likely a parasite sensor that is required for entry in mouse macrophages for some *T*. *cruzi* strains.

Moreover, the different patterns of gene expression observed in heat maps clearly differentiated between the three phases of macrophage infection. Interestingly, *Il1b* / *Il6* / *Irg1* expression increased at 24 hpi after infection in BALB/c macrophages infected with all the strains, except for VFRA. *Tnf* gene expression increased with infection, but in the absence of SLAMF1, higher levels of *Tnf* expression were observed at 1 and 6 hpi, decreasing at 24 hpi, suggesting that SLAMF1 might be retarding the induction of *Tnf* in infected macrophages with all the strains tested. In addition, *T*. *cruzi*-induced *Irg1* expression was lower in the absence of SLAMF1 at 1 h, but increased thereafter, in particular with the Y strain. *Irg1*, despite being a marker of M1, has been described to inhibit *Tnf* expression in macrophages [20]. Thus, it makes sense that at 1h lower levels of *Irg1* expression, could allow increased expression of *Tnf*. *Irg1* is highly expressed in mammalian macrophages and produces itaconic acid, which has an antimicrobial effect in macrophages inhibiting isocitrate lyase, the key enzyme of the glyoxylate shunt [21]. Interestingly, itaconic acid could eventually be useful for treating *T*. *cruzi* infection since itaconic acid might regulate MDSC-associated ROS production and MDSC-suppressive potential [22], which are host-detrimental in our model involving BALB/c mice and the Y parasite strain [16, 23, 24].

We found important differences regarding *Cybb* expression and ROS production by macrophages, with two major types of divergent responses to parasite strains. *Cybb* expression and ROS production increased with Dm28, Y, M6421, 10R26 and Bug2148, and decreased with VFRA.

It has been described that SLAMF1 positively regulates ROS production by NOX2 and phagosome maturation in response to bacterial infection [8]. On the other hand, production of ROS by NOX2 after endotoxin stimulation in macrophages takes place within minutes due to NOX2 basal level of expression [25]. Thus, ROS could be detected very early after infection and we found it detectable at 6 hpi when all the parasites likely have been already internalized.

However, ROS can be also triggered by several monocyte receptors besides SLAMF1, including TLRs, that are *T*. *cruzi* sensors [26] and also involved in internalization [27], suggesting that in *T*. *cruzi* infection by strains other than VFRA, interaction of parasite molecules with SLAMF1 may negatively regulate ROS production, by sequestering or interfering with normal activating functions of SLAMF1 via PI3P/NOX2. SLAMF1 affects two important phagosomal processes: NOX2 activation and the recruitment of EEA1, involved in phagolysosomal fusion. *T*. *cruzi* requires phagolysosome formation for establishing the infection [28]. Nevertheless, M6421 strain, despite being similar to VFRA regarding interaction, showed higher levels of *Cybb*, but slightly lower ROS production in *Slamf1*^*-/-*^ macrophages. This could be explained if M6421 strain, despite triggering *Cybb* expression, it did not trigger other NOX2 subunits needed for its activity and/or other receptors needed for ROS production. In general, we found that we high levels of ROS associate with a decreased parasite proliferation and with the involvement of SLAMF1.

In addition, it has been described that the SLAM family of receptors can be activators or inhibitors of immune cells, mostly depending on the expression of adaptors as SAP in immune cells [29] and phosphatases [30]. Thus another possibility to explain the differences in ROS production could be that infection affects the expression of adaptors in macrophages in a parasite strain-dependent manner.

Our results also suggest that VFRA may not strictly require SLAMF1 for interaction and internalization as Dm28, Y, 10R26 and Bug2148 strains did. However, VFRA was able to trigger more efficiently ROS production than the others, in a SLAMF1-dependent manner, since *Slamf1*^*-/-*^ macrophages showed a drop in *Cybb* expression and ROS production, indicating a functional interaction with SLAMF1, although of the opposite sign.

Taken together, we found an inverse correlation between ROS production and parasite replication. It has been described using other mouse models that ROS-derived signal is required for parasite proliferation [31–33]. However, these differences could be attributed to the use of different host and parasite strains in the experiments. Goes et al. found a possible explanation for this discrepancy; parasites need minimal levels of ROS, which would work as a signal for replication, but high levels of ROS are deleterious to the parasite, inducing, for example, DNA damage [31]. This highlights the importance of the background of the host in the outcome of the infection [2, 34]. Interestingly, by proteomic analysis, we have reported enriched antioxidant defenses in trypomastigotes of VFRA compared to Y strain [35], that could support the hypothesis that ROS production is detrimental for VFRA and for this reason VFRA would tend to increase these metabolic pathways to cope with the great generation of ROS by NOX2 among other enzymes.

The information obtained from macrophage infection allowed us to identify two strong divergent behaviors which fit with our previous reports [3, 13]. Thus, we selected the two strains representative of the two major behaviors, Y and VFRA, for *in vivo* studies, as well as Dm28 since it showed highest parasite load in macrophages. We were able to detect parasites and inflammatory infiltrates in all conditions, even in hearts of SLAMF1 deficient mice with both Y and VFRA strains. Surprisingly, Dm28 strain produced a very mild infection in mice, 100% survival, no parasitemia and very low number of parasites in spleen, liver and heart. The results reflect the discrepancies between in *vitro* and *in vivo* infectivity and put a word of caution of using only a single parasite strain for all kind of experimental Chagas disease studies including immunopathogenesis.

SLAMF1 deficiency differently affected the disease outcome depending on the strain. Parasitemia was lower in *Slamf1*^*-/-*^ than in BALB/c mice for Y and VFRA strains, despite VFRA being more infective in SLAMF1 deficient macrophages. In *Slamf1*^*-/-*^ mice infected with Y strain, parasite load decreased in spleen and liver compared to BALB/c. In the case of VFRA, the absence of SLAMF1 did not significantly affect spleen parasite load but increased it in the liver at the EA phase, suggesting that SLAMF1 plays a very important role in the liver for the control of parasite replication. Interestingly, a similar but milder effect was observed in the heart at the EA phase. More importantly, SLAMF1 deficiency abolished Y strain-induced mortality in BALB/c as previously described [6]. Dm28 *in vivo* infection, although much milder, was able to increase gene expression of several immune-related genes in heart tissue.

SLAMF1 may also affect the immune responses [8]. The immune/inflammatory response in intestine with Y and VFRA strains was very different. In the intestine BALB/c mice the infection with Y increased *Ifng / Il6* expression, but with VFRA there was an increased in *Cd4* / *Tnf* which was protective. SLAMF1 deficiency allowed the development of a similar protective response in mice infected with the Y strain with increase expression of *Cd4* / *Tnf*, and also *Cd11c / Cybb / Nos2*, likely because of stronger CD4 T cell and dendritic cell responses, respectively.

We previously reported that in heart tissue of susceptible BALB/c mice infected with the Y strain there was a lower Th1/Th2 balance [16], higher levels of *Il17* (Th17 marker) and lower levels of Foxp3 (Treg marker) than non-susceptible C57BL/6 mice [19]. In addition, in the susceptible model there was a host-detrimental expansion of M-MDSCs [16, 23, 24]. Here we found a detrimental increase in *Cd4 / Il6 / Il10* expression in heart tissue of BALB/c mice infected with the Y strain, not observed in the absence of SLAMF1, in which *Cd8* was increased at the LA phase likely allowing a better control of the infection. Moreover, myeloid markers also varied in the absence of SLAMF1, there was a decrease in *Arg1* and *S100a9*, suggesting lower levels of M-MDSCs and G-MDSCs, but increase in *Irg1 / Nos2* expression suggesting a shift towards M1 macrophage polarization.

There was no strict correlation between the parasitemia and macrophage parasite load in infections *in vitro* with Y and VFRA strains. These differences are not surprising since parasitemia reflects overall infection of different nucleated cells and tissues target of the infection that do not express SLAMF1. Thus, as we previously described, SLAMF1 was crucial for Y strain infecting macrophages and DCs but not for infecting cardiomyocytes *in vitro*, which do not express SLAMF1 in the cell surface [6].

In conclusion, our results indicate an important role of SLAMF1 interaction with *T*. *cruzi* infection that affects NOX2 expression and ROS production in immune cells in a parasite-strain-dependent manner and regulates immune responses. Although the ultimate cause of these differences between parasite strains remains not completely understood, differential expression by parasite strains of *T*. *cruzi* molecules interacting with SLAMF1 may explain it. Besides, the resistance of parasites to ROS and different proliferative rates, individually or more likely in combination, could explain these variations at the various sites and stages of infection. Thus, in BALB/c mice parasite molecules can either enhance or inhibit T cell activation by interfering homophilic SLAMF1 interactions of T cells and antigen-presenting cells. In the intestine, there is a high percentage of resident immune cells that can quickly respond to infection and mobilize more cells to this site. Is in the intestine where we can find increased expression NOX2 in the absence of SLAMF1 as it does in macrophages *in vitro* after infection with the Y strain. In addition, the intestine is close to the site of infection and is likely the first organ to respond and determine the parasite load and immune response in other organs as the heart. The *in vivo* study was limited to three parasite strains and it will be worthwhile to investigate the role of SLAMF1 *in vivo* with more strains. Taken together, our results emphasize the diversity of the pathogenicity of *T*. *cruzi* strains and reinforce the need for studying several parasite strains to fully address the role of SLAMF1 in the immunopathogenesis of the disease.

## Supporting information

S1 Table Gene primer sequences. Forward (f) and reverse (r) sets of primers are indicated.
(DOCX)

S2 Table Principal component analysis of gene expression in BALB/c and *Slamf1^-/-^* macrophages. Analysis was performed using R software and mean values of gene expression.
(DOCX)

S3 Table Gene expression in intestine of BALB/c and *Slamf1^-/-^* mice infected with Y and VFRA strains of *T*. *cruzi*. Mean and standard deviation of RQ values corresponding to 5 mice per group (n = 5) as described in the materials and methods section.
(DOCX)

S4 Table Principal component analysis of gene expression in intestine of BALB/c and *Slamf1^-/-^* mice. Analysis was performed using R software and mean values of gene expression.
(DOCX)

S5 Table Gene expression in heart tissue of BALB/c and *Slamf1^-/-^* mice infected with Y and VFRA strains of *T*. *cruzi*. Mean and standard deviation of RQ values corresponding to 5 mice per group (n = 5) as described in the materials and methods section.
(DOCX)

S6 Table Principal component analysis of gene expression in heart tissue of BALB/c and *Slamf1^-/-^* mice. Analysis was performed using R software and mean values of gene expression.
(DOCX)

S1 Fig mRNA gene expression in BALB/c and *Slamf1^-/-^* macrophages infected with Dm28, Y, M6421, 10R26, Bug2841, and VFRA strains of *T*. *cruzi*. RTqPCR was performed as described in Methods. The Graphs represent the mean ± SEM (n = 6) calculated as RQ values corresponding to 5 mice per group (n = 5) as described in the materials and methods section. The asterisks indicate the statistical significance after t student test (*p<0.05. **p<0.005 and ***p<0.001) between BALB/c and *Slamf1^-/-^* macrophages.
(TIF)

S2 Fig Basal mRNA expression of genes in non-infected peritoneal macrophages. RTqPCR was performed as described in Methods. The bar graphs represent mean ΔCt values in BALB/c and *Slamf1*^*-/-*^ non-infected macrophages (n = 6).
(TIF)

S3 Fig Splenomegaly and parasite load in spleen of BALB/c and *Slamf1*^*-/-*^ mice infected with Dm28. Y and VFRA strains of *T*. *cruzi*. A) Splenomegaly at the EA phase. B) Splenomegaly at the LA phase. C) Parasite load at the EA phase. D) Parasite load at the LA phase. Mean ± SEM of 5 mice per group is represented. The asterisks indicate the statistical significance after t student test (*p<0.05. **p<0.005 and ***p<0.001). when comparing BALB/c and *Slamf1*^*-/-*^ infected mice. Asterisks alone stand for differences to mice that are not infected and horizontal bars and asterisks correspond to comparisons between BALB/c and *Slamf1*^*-/-*^ mice.
(TIF)

S4 Fig Hepatomegaly and parasite load of BALB/c and *Slamf1^-/-^* mice infected with Dm28, Y and VFRA strains of *T*. *cruzi*. A) Hepatomegaly at the EA phase. B) Hepatomegaly at the LA phase. C) Parasite load at the EA phase. D) Parasite load at the LA phase. Mean ± SEM of 5 mice per group is represented. The asterisks indicate the statistical significance after t student test (*p<0.05. **p<0.005 and ***p<0.001). when comparing the infected BALB/c and *Slamf1*^*-/-*^ mice. Asterisks alone stand for differences to mice that are not infected and horizontal bars and asterisks correspond to comparisons between BALB/c and *Slamf1*^*-/-*^ mice.
(TIF)

S5 Fig Parasite load in the intestine in BALB/c and *Slamf1*^*-/-*^ mice infected with Y and VFRA strains of *T*. *cruzi*. A) EA phase. B) LA phase. Mean ± SEM of 5 mice per group is represented. The asterisks indicate the statistical significance after t student test (*p<0.05. **p<0.005 and ***p<0.001). Asterisks alone stand for differences to mice that are not infected and horizontal bars and asterisks correspond to comparisons either between BALB/c and *Slamf1*^*-/-*^ mice.
(TIF)

S6 Fig Heart hypertrophy and parasite load in BALB/c and *Slamf1*^*-/-*^ mice infected with Dm28, Y and VFRA strains of *T*. *cruzi*. A) Hypertrophy at the EA phase. B) Hypertrophy at the LA phase. C) Parasite load at the EA phase. D) Parasite load at the LA phase. Mean ± SEM of 5 mice per group is represented. The asterisks indicate the statistical significance after t student test (*p<0.05. **p<0.005 and ***p<0.001). Asterisks alone stand for differences to mice that are not infected and horizontal bars and asterisks correspond to comparisons between BALB/c and *Slamf1*^*-/-*^ mice.
(TIF)

S7 Fig Hematoxylin & Eosin staining of cardiac tissue of BALB/c and *Slamf1*^*-/-*^ mice infected with the Dm28. **Y and VFRA strains of *T*. *cruzi* at EA phase.** Hematoxylin & Eosin staining are shown. Arrows indicate amastigote nests.
(TIF)

S8 Fig mRNA gene expression in heart of BALB/c and *Slamf1*^*-/-*^ mice infected with Dm28 strain of *T*. *cruzi*. The Graphs represent the mean ± SEM (n = 5) calculated as RQ values corresponding to 5 mice per group (n = 5) as described in the materials and methods section. The asterisks indicate the statistical significance after t student test (*p<0.05. **p<0.005 and ***p<0.001) between BALB/c and *Slamf1*^*-/-*^ mice.
(TIF)
