# Supplementary material for: Interaction of Signaling Lymphocytic Activation Molecule Family 1 (SLAMF1) receptor with Trypanosoma cruzi is strain-dependent and affects NADPH oxidase expression and activity
Source: PLoS Negl Trop Dis. 2020 Sep 14;14(9):e0008608. doi: 10.1371/journal.pntd.0008608 (PMC7515593; doi:10.1371/journal.pntd.0008608)
Supplement: S1 Table — Forward (f) and reverse (r) sets of primers are indicated. (DOCX) [file pntd.0008608.s001.docx]

**S1 Table. Gene primer sequences.** Forward (*f*) and reverse (*r*) sets of primers are indicated.

| **Gene** | **Primer sequence** |
| --- | --- |
|  | ***Mus musculus*** |
| *18s f* | 5’-GCAATTATTCCCCATGAACG-3’ |
| *18s r* | 5’-GGGACTTAATCAACGCAAGC-3’ |
| *Arg1 f* | 5’-AGAGATTATCGGAGCGCCTT-3’ |
| *Arg1 r* | 5’-TTTTTCCAGCAGACCAGCTT-3’ |
| *Cd11c f* | 5’-CTGGATAGCCTTTCTTCTGCTG-3’ |
| *Cd11c r* | 5’-GCACACTGTGTCCGAACTC-3’ |
| *Cd206 f* | 5’-CTCTGTTCAGCTATTGGACGC-3’ |
| *Cd206 r* | 5’-CGGAATTTCTGGGATTCAGCTTC-3’ |
| *Cd4 f* | 5’-CACCTGTGCAAGAAGCAGAG-3’ |
| *Cd4 r* | 5’-CAAGCGCCTAAGAGAGATGG-3’ |
| *Cd68 f* | 5’-TTCAGGGTGGAAGAAAGGTAAAGG-3’ |
| *Cd68r* | 5’-CAATGATGAGAGGCAGCAAGAGG-3’ |
| *Cd8 f* | 5’-ACAGGGACGAAGCTGACTGT-3’ |
| *Cd8 r* | 5’-ACGGGCATTGCTTGTTGTT-3’ |
| *Cybb f* | 5’-CTTTCTCAGGGGTTCCAGTG-3’ |
| *Cybb r* | 5’-TGCAGTGCTATCATCCAAGC-3’ |
| *Foxp3 f* | 5’-TGGCAGAGAGGTATTGAGGG-3’ |
| *Foxp3 r* | 5’-CTCGTCTGAAGGCAGAGTCA-3’ |
| *Ifng f* | 5’-ACAGCAAGGCGAAAAAGGAT-3’ |
| *Ifng r* | 5’-TGAGCTCATTGAATGCTTGG-3’ |
| *Il10 f* | 5’-ATCGATTTCTCCCCTGTGAA-3’ |
| *Il10 r* | 5’-TGTCAAATTCATTCATGGCCT-3’ |
| *Il13 f* | 5’-TGTGTCTCTCCCTCTGACCC-3’ |
| *Il13 r* | 5’-CACACTCCATACCATGCTGC-3’ |
| *Il17 f* | 5’-TCCAGAAGGCCCTCAGACTA-3’ |
| *Il17 r* | 5’-TGAGCTTCCCAGATCACAGA-3’ |
| *Il1b f* | 5’-TGTGAAATGCCACCTTTTGA-3’ |
| *Il1b r* | 5’-GGTCAAAGGTTTGGAAGCAG-3’ |
| *IL4r f* | 5’-ACAGCGCACCACACTGACACT-3’ |
| *IL4r r* | 5’-CTGGCACCTGGAGTGAGTGG-3’ |
| *Il6 f* | 5’-TGATGCACTTGCAGAAAACA-3’ |
| *Il6 r* | 5’-ACCAGAGGAAATTTTCAATAGGC-3’ |
| *Irg1 f* | 5’-CCTGTGCCTCGCTGCTCGAC-3’ |
| *Irg1 r* | 5’-CGTGTCGAAGCTTGGCGGGT-3’ |
| *Nos2 f* | 5’-TGAAGAAAACCCCTTGTGCT-3’ |
| *Nos2 r* | 5’-TTCTGTGCTGTCCCAGTGAG-3’ |
| *Ptges f* | 5’-GATCTCCTGGCTGCAAA-3’ |
| *Ptges r* | 5’-CCTGGACAGTGCTTTGCTC-3’ |
| *S100a9 f* | 5’-TCAGACAAATGGTGGAAGCA-3’ |
| *S100a9 r* | 5’-GTCCAGGTCCTCCATGATGT-3’ |
| *Tgfb f* | 5’-GGAGAGCCCTGGATACCAAC-3’ |
| *Tgfb r* | 5’-CAACCCAGGTCCTTCCTAAA-3’ |
| *Tnf f* | 5’-CCACCACGCTCTTCTGTCTAC-3’ |
| *Tnf r* | 5’-AGGGTCTGGGCCATAGAACT-3’ |
|  | ***Homo sapiens*** |
| *18S f* | 5’-GCAATTATTCCCCATGAACG-3’ |
| *18S r* | 5’-GGGACTTAATCAACGCAAGC-3’ |
| *SLAMF1f* | 5’-GAGCATGCGCATGATGAACTGCCCAAAG-3’ |
| *SLAMF1r* | 5’-CCAGATCTTGAGGGGTCTGTCCTGGATCC-3’ |
