# Supplementary material for: Interaction of Signaling Lymphocytic Activation Molecule Family 1 (SLAMF1) receptor with Trypanosoma cruzi is strain-dependent and affects NADPH oxidase expression and activity
Source: PLoS Negl Trop Dis. 2020 Sep 14;14(9):e0008608. doi: 10.1371/journal.pntd.0008608 (PMC7515593; doi:10.1371/journal.pntd.0008608)
Supplement: S2 Table — Analysis was performed using R software and mean values of gene expression. (DOCX) [file pntd.0008608.s002.docx]

**S2 Table. Principal component analysis of gene expression in BALB/c and *Slamf1 ^-/-^* macrophages.** Analysis was performed using R software and mean values of gene expression.

| **BALB/c** | Importance of components: |  |  |  |  |  |  |  |
| --- | --- | --- | --- | --- | --- | --- | --- | --- |
|  |  | PC1 | PC2 | PC3 | PC4 | PC5 | PC6 | PC7 |
|  | Standard deviation | 1.470 | 1.122 | 0.598 | 0.363 | 0.251 | 0.167 | 0.135 |
|  | Proportion of Variance | 0.538 | 0.313 | 0.089 | 0.033 | 0.016 | 0.007 | 0.005 |
|  | Cumulative Proportion | 0.538 | 0.851 | 0.940 | 0.973 | 0.989 | 0.996 | 1.000 |
|  | Contribution |  |  |  |  |  |  |  |
|  |  | Dim.1 | Dim.2 | Dim.3 | Dim.4 | Dim.5 | Dim.6 | Dim.7 |
|  | *Cybb* | 12.721 | 71.504 | 14.859 | 0.437 | 0.059 | 0.349 | 0.071 |
|  | *Arg1* | 0.210 | 9.103 | 37.532 | 2.759 | 3.440 | 24.389 | 22.566 |
|  | *Il10* | 0.013 | 5.134 | 42.200 | 1.782 | 12.760 | 18.535 | 19.575 |
|  | *Tnf* | 0.125 | 2.140 | 3.358 | 77.596 | 7.340 | 1.915 | 7.526 |
|  | *Il6* | 36.394 | 4.020 | 1.822 | 2.222 | 3.862 | 31.272 | 20.408 |
|  | *Irg1* | 20.831 | 3.193 | 0.043 | 4.142 | 56.408 | 15.055 | 0.328 |
|  | *Il1b* | 29.705 | 4.905 | 0.185 | 11.062 | 16.131 | 8.484 | 29.527 |

| ***Slamf1^-/-^*** | Importance of components: |  |  |  |  |  |  |  |
| --- | --- | --- | --- | --- | --- | --- | --- | --- |
|  |  | PC1 | PC2 | PC3 | PC4 | PC5 | PC6 | PC7 |
|  | Standard deviation | 2.177 | 1.243 | 0.863 | 0.593 | 0.277 | 0.212 | 0.109 |
|  | Proportion of Variance | 0.631 | 0.206 | 0.099 | 0.047 | 0.010 | 0.006 | 0.002 |
|  | Cumulative Proportion | 0.631 | 0.836 | 0.935 | 0.982 | 0.992 | 0.998 | 1.000 |
|  | Contribution |  |  |  |  |  |  |  |
|  |  | Dim.1 | Dim.2 | Dim.3 | Dim.4 | Dim.5 | Dim.6 | Dim.7 |
|  | *Cybb* | 16.487 | 19.169 | 58.998 | 1.362 | 2.469 | 0.462 | 1.053 |
|  | *Arg1* | 1.897 | 0.507 | 0.823 | 60.520 | 10.252 | 18.001 | 7.999 |
|  | *Il10* | 0.079 | 3.140 | 3.031 | 31.062 | 35.027 | 7.541 | 20.120 |
|  | *Tnf* | 12.663 | 47.600 | 35.274 | 0.635 | 0.308 | 3.463 | 0.057 |
|  | *Il6* | 44.150 | 13.102 | 1.393 | 1.956 | 7.525 | 4.697 | 27.177 |
|  | *Irg1* | 15.354 | 6.853 | 0.381 | 2.436 | 37.505 | 8.638 | 28.834 |
|  | *Il1b* | 9.370 | 9.630 | 0.100 | 2.029 | 6.913 | 57.197 | 14.761 |
