# Supplementary material for: Interaction of Signaling Lymphocytic Activation Molecule Family 1 (SLAMF1) receptor with Trypanosoma cruzi is strain-dependent and affects NADPH oxidase expression and activity
Source: PLoS Negl Trop Dis. 2020 Sep 14;14(9):e0008608. doi: 10.1371/journal.pntd.0008608 (PMC7515593; doi:10.1371/journal.pntd.0008608)
Supplement: S3 Table — Mean and standard deviation of RQ values corresponding to 5 mice per group (n = 5) as described in the materials and methods section. (DOCX) [file pntd.0008608.s003.docx]

**S3 Table. Gene expression in intestine of BALB/c and *Slamf1^-/-^* mice infected with Y and VFRA strains of *T. cruzi*.** Mean and standard deviation of RQ values corresponding to 5 mice per group (n=5) as described in the materials and methods section.

| **BALB/c** | **Lymphoid Markers** | | | | | | | |
| --- | --- | --- | --- | --- | --- | --- | --- | --- |
|  | **Y_EA** | | **VFRA_EA** | | **Y_LA** | | **VFRA_LA** | |
|  | Mean | SD | Mean | SD | Mean | SD | Mean | SD |
| *Il6* | 2.13 | 2.25 | 0.1 | 0.1 | 4.21 | 2.26 | 0.004 | 0.004 |
| *Il13* | 2.61 | 1.99 | 6.43 | 4.9 | 0.26 | 0.12 | 5.06 | 3.13 |
| *Tgfb* | 0.86 | 0.77 | 1.02 | 1 | 4.97 | 0.53 | 0.92 | 0.12 |
| *Tnf* | 6.2 | 4.88 | 137 | 150 | 3.21 | 0.13 | 47.9 | 9.14 |
| *Cd4* | 5.34 | 0.72 | 123.3 | 56.92 | 1.53 | 1.17 | 11.91 | 5.25 |
| *Foxp3* | 3.73 | 1.74 | 3.3 | 1.23 | 122.59 | 69.63 | 9.22 | 4.26 |
| *Ifng* | 808.73 | 506.71 | 4.98 | 1.27 | 0.47 | 0.28 | 26.01 | 5.14 |
| *Cd8* | 17.37 | 21.55 | 5.8 | 1.14 | 1.09 | 0.36 | 0.57 | 0.41 |
| *Il10* | 18.57 | 8.78 | 8.49 | 3.6 | 41.39 | 9.29 | 8.82 | 1.39 |
|  |  |  |  |  |  |  |  |  |
|  | **Myeloid Markers** | | | | | | | |
|  | **Y_EA** | | **VFRA_EA** | | **Y_LA** | | **VFRA_LA** | |
|  | Mean | SD | Mean | SD | Mean | SD | Mean | SD |
| *Irg1* | 0.12 | 0.12 | 0.009 | 0.006 | 7.77 | 3.8 | 0.04 | 0.03 |
| *Cd68* | 0.08 | 0.08 | 2.23 | 2.7 | 0.03 | 0.02 | 1.31 | 1.54 |
| *Il4r* | 11.44 | 7.19 | 0.31 | 0.13 | 4.97 | 0.18 | 1.25 | 1.33 |
| *Cd206* | 8.42 | 1.44 | 12.45 | 4.81 | 811.82 | 624.91 | 14.74 | 10.15 |
| *Ptges* | 148.11 | 11.85 | 1.98 | 1.39 | 1.55 | 1.25 | 53.2 | 27.83 |
| *Cd11c* | 12.77 | 1.35 | 6.79 | 1.8 | 29.61 | 20.99 | 5.1 | 2.76 |
| *Cybb* | 1.01 | 0.94 | 3.84 | 1.23 | 0.43 | 0.42 | 0.75 | 0.46 |
| *Arg1* | 2.73 | 1.79 | 4.36 | 0.89 | 11.72 | 3.79 | 2.5 | 1.07 |
| *S100a9* | 48.38 | 33.2 | 3.38 | 0.88 | 106.6 | 88.9 | 2.96 | 1.52 |
| *Nos2* | 18.43 | 14.59 | 55.06 | 30.94 | 7.43 | 4.17 | 29.78 | 9.62 |

| ***Slamf1^-/-^*** | **Lymphoid Markers** | | | | | | | |
| --- | --- | --- | --- | --- | --- | --- | --- | --- |
|  | **Y_EA** | | **VFRA_EA** | | **Y_LA** | | **VFRA_LA** | |
|  | Mean | SD | Mean | SD | Mean | SD | Mean | SD |
| *Il6* | 232.75 | 205.56 | 139.92 | 38.28 | 60.23 | 32.5 | 12.93 | 3.05 |
| *Il13* | 7.37 | 6.07 | 9.19 | 6.19 | 11.08 | 8.92 | 11.86 | 6.23 |
| *Tgfb* | 5.99 | 2.22 | 22.87 | 8.93 | 12.51 | 7.08 | 5.87 | 2.6 |
| *Tnf* | 16.27 | 1 | 1.16 | 0.7 | 6.2 | 3.11 | 5.46 | 1.27 |
| *Cd4* | 53.68 | 29.96 | 0.91 | 0.29 | 6.04 | 3.32 | 1.75 | 0.36 |
| *Foxp3* | 18.89 | 11.18 | 258.86 | 194.1 | 128.36 | 73.98 | 21.11 | 7.46 |
| *Ifng* | 1425.57 | 1099.58 | 124.31 | 26.53 | 2923.06 | 374.31 | 257.15 | 131.49 |
| *Cd8* | 301.34 | 134.93 | 45.79 | 8.32 | 389.56 | 381.22 | 175.98 | 71.86 |
| *Il10* | 1142.02 | 200.2 | 100.6 | 15.33 | 154.69 | 28.73 | 41.62 | 7.17 |
|  |  |  |  |  |  |  |  |  |
|  | **Myeloid Markers** | | | | | | | |
|  | **Y_EA** | | **VFRA_EA** | | **Y_LA** | | **VFRA_LA** | |
|  | Mean | SD | Mean | SD | Mean | SD | Mean | SD |
| *Irg1* | 490.07 | 257.69 | 38.29 | 2.52 | 215.43 | 21.14 | 90.25 | 5.89 |
| *Cd68* | 44.83 | 22.1 | 7.91 | 5.24 | 10.81 | 7.75 | 65.62 | 39.83 |
| *Il4r* | 74.79 | 69.26 | 1.82 | 0.73 | 4.8 | 3.96 | 12.14 | 4.95 |
| *Cd206* | 11.29 | 3.22 | 6.78 | 1.53 | 1.29 | 0.25 | 9.18 | 1.91 |
| *Ptges* | 15.32 | 1.4 | 15.52 | 4.29 | 7.6 | 2.31 | 72.88 | 14.03 |
| *Cd11c* | 502.07 | 125.97 | 127.64 | 26.57 | 9.73 | 3.66 | 13.71 | 2.5 |
| *Cybb* | 7306.2 | 2319.33 | 24.65 | 6.6 | 77.99 | 20.53 | 14.24 | 3.91 |
| *Arg1* | 156.82 | 85.84 | 30.03 | 9.12 | 221.79 | 119.51 | 54.63 | 30.88 |
| *S100a9* | 1031.64 | 206.71 | 67.23 | 32.8 | 1978.95 | 963.53 | 108.62 | 70.18 |
| *Nos2* | 905.9 | 856.54 | 134.17 | 46.34 | 449.54 | 208.15 | 34.01 | 16.67 |
