# Supplementary material for: Interaction of Signaling Lymphocytic Activation Molecule Family 1 (SLAMF1) receptor with Trypanosoma cruzi is strain-dependent and affects NADPH oxidase expression and activity
Source: PLoS Negl Trop Dis. 2020 Sep 14;14(9):e0008608. doi: 10.1371/journal.pntd.0008608 (PMC7515593; doi:10.1371/journal.pntd.0008608)
Supplement: S4 Table — Analysis was performed using R software and mean values of gene expression. (DOCX) [file pntd.0008608.s004.docx]

**S4 Table. Principal component analysis of gene expression in intestine of BALB/c and *Slamf1 ^-/-^* mice.** Analysis was performed using R software and mean values of gene expression.

| **Lymphoid BALB/c** | Importance of components: |  |  |  |  |
| --- | --- | --- | --- | --- | --- |
|  |  | PC1 | PC2 | PC3 | PC4 |
|  | Standard deviation | 1.883 | 1.513 | 0.887 | 0.000 |
|  | Proportion of Variance | 0.536 | 0.346 | 0.119 | 0.000 |
|  | Cumulative Proportion | 0.536 | 0.881 | 1.000 | 1.000 |
|  | Contribution |  |  |  |  |
|  |  | Dim.1 | Dim.2 | Dim.3 | Dim.4 |
|  | *Il6* | 35.698 | 21.294 | 20.891 | 17.269 |
|  | *Il13* | 11.096 | 0.104 | 1.079 | 1.192 |
|  | *Tgfb* | 3.228 | 0.817 | 0.002 | 0.008 |
|  | *Tnf* | 11.360 | 4.273 | 9.680 | 21.432 |
|  | *Cd4* | 10.721 | 1.120 | 30.351 | 0.593 |
|  | *Foxp3* | 10.977 | 4.408 | 5.323 | 2.460 |
|  | *Ifng* | 13.894 | 53.872 | 15.723 | 3.661 |
|  | *Cd8* | 0.186 | 13.975 | 16.746 | 27.349 |
|  | *Il10* | 2.839 | 0.138 | 0.206 | 26.036 |

| **Myeloid BALB/c** | Importance of components: |  |  |  |  |
| --- | --- | --- | --- | --- | --- |
|  |  | PC1 | PC2 | PC3 | PC4 |
|  | Standard deviation | 2.044 | 1.222 | 0.482 | 0.000 |
|  | Proportion of Variance | 0.708 | 0.253 | 0.039 | 0.000 |
|  | Cumulative Proportion | 0.708 | 0.961 | 1.000 | 1.000 |
|  | Contribution |  |  |  |  |
|  |  | Dim.1 | Dim.2 | Dim.3 | Dim.4 |
|  | *Irg1* | 37.375 | 0.046 | 11.266 | 9.234 |
|  | *Cd68* | 17.395 | 5.340 | 11.340 | 25.093 |
|  | *Il4r* | 5.426 | 16.356 | 1.465 | 0.265 |
|  | *Cd206* | 15.820 | 11.607 | 12.432 | 0.344 |
|  | *Ptges* | 1.877 | 60.084 | 12.224 | 0.115 |
|  | *Cd11c* | 2.537 | 0.015 | 3.645 | 4.972 |
|  | *Cybb* | 2.326 | 1.063 | 21.546 | 2.615 |
|  | *Arg1* | 1.369 | 2.427 | 0.548 | 10.460 |
|  | *S100a9b* | 12.785 | 2.822 | 24.621 | 23.901 |
|  | *Nos2* | 3.090 | 0.239 | 0.913 | 23.002 |

| **Lymphoid *Slamf1^-/-^*** | Importance of components: |  |  |  |  |
| --- | --- | --- | --- | --- | --- |
|  |  | PC1 | PC2 | PC3 | PC4 |
|  | Standard deviation | 1.280 | 0.756 | 0.534 | 0.000 |
|  | Proportion of Variance | 0.657 | 0.229 | 0.114 | 0.000 |
|  | Cumulative Proportion | 0.657 | 0.886 | 1.000 | 1.000 |
|  | Contribution |  |  |  |  |
|  |  | Dim.1 | Dim.2 | Dim.3 | Dim.4 |
|  | *Il6* | 3.320 | 41.750 | 2.958 | 18.700 |
|  | *Il13* | 0.164 | 0.624 | 0.815 | 16.276 |
|  | *Tgfb* | 1.791 | 7.377 | 3.162 | 20.007 |
|  | *Tnf* | 11.692 | 5.838 | 0.081 | 5.425 |
|  | *Cd4* | 36.620 | 0.580 | 1.629 | 14.559 |
|  | *Foxp3* | 6.543 | 24.231 | 27.226 | 2.084 |
|  | *Ifng* | 16.259 | 0.151 | 48.630 | 0.002 |
|  | *Cd8* | 6.678 | 5.460 | 10.972 | 22.382 |
|  | *Il10* | 16.933 | 13.989 | 4.527 | 0.566 |

| **Myeloid *Slamf1^-/-^*** | Importance of components: |  |  |  |  |
| --- | --- | --- | --- | --- | --- |
|  |  | PC1 | PC2 | PC3 | PC4 |
|  | Standard deviation | 1.713 | 0.991 | 0.771 | 0.000 |
|  | Proportion of Variance | 0.651 | 0.218 | 0.132 | 0.000 |
|  | Cumulative Proportion | 0.651 | 0.868 | 1.000 | 1.000 |
|  | Contribution | |  |  |  |
|  |  | Dim.1 | Dim.2 | Dim.3 | Dim.4 |
|  | *Irg1* | 6.154 | 1.862 | 4.914 | 54.524 |
|  | *Cd68* | 0.521 | 4.322 | 24.697 | 9.730 |
|  | *Il4r* | 10.406 | 3.512 | 22.309 | 5.269 |
|  | *Cd206* | 0.197 | 17.759 | 0.620 | 3.219 |
|  | *Ptges* | 0.987 | 7.194 | 12.168 | 2.685 |
|  | *Cd11c* | 9.666 | 24.753 | 22.730 | 3.084 |
|  | *Cybb* | 51.210 | 1.172 | 0.265 | 10.386 |
|  | *Arg1* | 2.457 | 7.729 | 2.284 | 8.642 |
|  | *S100a9* | 8.491 | 26.384 | 1.829 | 0.903 |
|  | *Nos2* | 9.911 | 5.312 | 8.185 | 1.559 |
