# Supplementary material for: Interaction of Signaling Lymphocytic Activation Molecule Family 1 (SLAMF1) receptor with Trypanosoma cruzi is strain-dependent and affects NADPH oxidase expression and activity
Source: PLoS Negl Trop Dis. 2020 Sep 14;14(9):e0008608. doi: 10.1371/journal.pntd.0008608 (PMC7515593; doi:10.1371/journal.pntd.0008608)
Supplement: S5 Table — Mean and standard deviation of RQ values corresponding to 5 mice per group (n = 5) as described in the materials and methods section. (DOCX) [file pntd.0008608.s005.docx]

**S5 Table. Gene expression in heart tissue of BALB/c and *Slamf1^-/-^* mice infected with Y and VFRA strains of *T. cruzi*.** Mean and standard deviation of RQ values corresponding to 5 mice per group (n=5) as described in the materials and methods section.

| **BALB/c** | **Lymphoid Markers** | | | | | | | |
| --- | --- | --- | --- | --- | --- | --- | --- | --- |
|  | **Y_EA** | | **VFRA_EA** | | **Y_LA** | | **VFRA_LA** | |
|  | Mean | SD | Mean | SD | Mean | SD | Mean | SD |
| *Foxp3* | 1.11 | 0.77 | 0.50 | 0.27 | 0.38 | 0.15 | 0.19 | 0.12 |
| *Il17* | 2.48 | 1.41 | 0.36 | 0.4 | 5.39 | 1.21 | 0.21 | 0.13 |
| *Tgfb* | 3.60 | 0.64 | 17.58 | 3.44 | 12.42 | 5.00 | 1.91 | 0.73 |
| *Tnf* | 14.51 | 0.81 | 4.02 | 1.40 | 111.65 | 73.46 | 0.61 | 0.54 |
| *Cd4* | 15.96 | 2.21 | 18.20 | 9.35 | 1.9 | 0.72 | 1.31 | 0.16 |
| *Il13* | 27.36 | 1.68 | 127.07 | 15.82 | 7.62 | 2.03 | 14.77 | 3.27 |
| *Cd8* | 98.21 | 31.49 | 12.72 | 12.37 | 96.34 | 53.68 | 105.54 | 43.38 |
| *Il10* | 328.83 | 0.73 | 179.13 | 1.44 | 32.80 | 19.30 | 4.10 | 1.93 |
| *Il6* | 952.84 | 0.46 | 76.87 | 1.11 | 19.06 | 14.71 | 15.14 | 8.93 |
| *Ifng* | 120.54 | 42.29 | 681.28 | 143.36 | 56.66 | 14.08 | 81.82 | 7.10 |
|  |  |  |  |  |  |  |  |  |
|  | **Myeloid Markers** | | | | | | | |
|  | **Y_EA** | | **VFRA_EA** | | **Y_LA** | | **VFRA_LA** | |
|  | Mean | SD | Mean | SD | Mean | SD | Mean | SD |
| *Ptges* | 1.90 | 1.22 | 0.28 | 0.28 | 2.38 | 0.95 | 0.27 | 0.14 |
| *Cd206* | 1.99 | 0.94 | 0.39 | 0.68 | 0.28 | 0.40 | 0.40 | 0.31 |
| *Cd68* | 102.13 | 17.18 | 30.46 | 11.32 | 8.89 | 5.78 | 17.34 | 4.02 |
| *Il4r* | 10.26 | 1.13 | 2.59 | 0.68 | 5.73 | 1.75 | 6.44 | 2.20 |
| *S100a9* | 48.66 | 5.98 | 5.45 | 6.30 | 47.49 | 7.98 | 1.34 | 1.97 |
| *Cybb* | 18.51 | 0.53 | 10.14 | 1.60 | 73.06 | 26.41 | 131.16 | 31.02 |
| *Irg1* | 796.79 | 0.63 | 93.50 | 1.42 | 11.02 | 0.60 | 2.51 | 1.13 |
| *Arg1* | 1768.22 | 0.45 | 452.39 | 1.01 | 69.54 | 9.88 | 217.64 | 112.16 |
| *Nos2* | 380.12 | 141.23 | 27.56 | 20.9 | 978.17 | 735.98 | 535.96 | 277.9 |

| ***Slamf1^-/-^*** | **Lymphoid Markers** | | | | | | | |
| --- | --- | --- | --- | --- | --- | --- | --- | --- |
|  | **Y_EA** | | **VFRA_EA** | | **Y_LA** | | **VFRA_LA** | |
|  | Mean | SD | Mean | SD | Mean | SD | Mean | SD |
| *Foxp3* | 0.91 | 0.26 | 0.34 | 0.11 | 0.44 | 0.04 | 1.38 | 0.10 |
| *Il17* | 20.76 | 16.4 | 1.02 | 0.5 | 16.56 | 12.82 | 0.83 | 0.39 |
| *Tgfb* | 3.45 | 0.54 | 1.04 | 0.42 | 3.11 | 0.36 | 7.28 | 1.75 |
| *Tnf* | 27.67 | 0.47 | 6.63 | 0.33 | 110.69 | 61.27 | 0.80 | 0.45 |
| *Cd4* | 5.36 | 3.82 | 2.07 | 0.49 | 8.76 | 1.38 | 7.11 | 5.32 |
| *Il13* | 5.46 | 1.47 | 1.06 | 0.31 | 2.63 | 0.81 | 7.15 | 1.61 |
| *Cd8* | 6.32 | 3.12 | 1 | 0.89 | 420.37 | 266.69 | 29.38 | 11.89 |
| *Il10* | 101.05 | 0.42 | 24.02 | 0.37 | 50.01 | 31.69 | 11.30 | 1.80 |
| *Il6* | 249.59 | 0.61 | 18.26 | 0.48 | 17.18 | 18.74 | 16.83 | 6.78 |
| *Ifng* | 64.91 | 11.92 | 19.45 | 1.08 | 86.39 | 14.31 | 115.27 | 5.33 |
|  |  |  |  |  |  |  |  |  |
|  | **Myeloid Markers** | | | | | | | |
|  | **Y_EA** | | **VFRA_EA** | | **Y_LA** | | **VFRA_LA** | |
|  | Mean | SD | Mean | SD | Mean | SD | Mean | SD |
| *Ptges* | 0.96 | 0.22 | 0.72 | 0.33 | 1.70 | 0.27 | 1.92 | 0.59 |
| *Cd206* | 8.73 | 3.12 | 5.33 | 1.00 | 4.28 | 0.53 | 18.61 | 4.09 |
| *Cd68* | 25.47 | 25 | 5.44 | 3.84 | 10.07 | 5.05 | 11.86 | 6.04 |
| *Il4r* | 23.02 | 2.21 | 11.71 | 2.95 | 27.72 | 4.35 | 26.02 | 5.78 |
| *S100a9* | 3.59 | 1.29 | 3.35 | 0.51 | 45.75 | 6.25 | 6.94 | 1.30 |
| *Cybb* | 22.97 | 0.42 | 8.43 | 0.54 | 33.87 | 11.03 | 54.14 | 22.81 |
| *Irg1* | 1033.67 | 0.30 | 158.46 | 0.59 | 20.95 | 13.16 | 4.27 | 1.51 |
| *Arg1* | 722.11 | 0.64 | 52.98 | 1.80 | 7.11 | 8.19 | 12.13 | 2.74 |
| *Nos2* | 1514 | 1072.17 | 68.56 | 53 | 1944.03 | 957.4 | 294.09 | 152.68 |
