# Supplementary material for: Interaction of Signaling Lymphocytic Activation Molecule Family 1 (SLAMF1) receptor with Trypanosoma cruzi is strain-dependent and affects NADPH oxidase expression and activity
Source: PLoS Negl Trop Dis. 2020 Sep 14;14(9):e0008608. doi: 10.1371/journal.pntd.0008608 (PMC7515593; doi:10.1371/journal.pntd.0008608)
Supplement: S6 Table — Analysis was performed using R software and mean values of gene expression. (DOCX) [file pntd.0008608.s006.docx]

**S6 Table. Principal component analysis of gene expression in heart tissue of BALB/c and *Slamf1 ^-/-^* mice.** Analysis was performed using R software and mean values of gene expression.

| **Lymphoid BALB/c** | Importance of components: |  |  |  |  |
| --- | --- | --- | --- | --- | --- |
|  |  | PC1 | PC2 | PC3 | PC4 |
|  | Standard deviation | 1.451 | 1.211 | 0.750 | 0.000 |
|  | Proportion of Variance | 0.510 | 0.355 | 0.136 | 0.000 |
|  | Cumulative Proportion | 0.510 | 0.864 | 1.000 | 1.000 |
|  | Contributions |  |  |  |  |
|  |  | Dim.1 | Dim.2 | Dim.3 | Dim.4 |
|  | *Foxp3* | 4.381 | 0.000 | 1.456 | 29.679 |
|  | *Il17* | 6.078 | 21.789 | 0.305 | 2.065 |
|  | *Tgfb* | 2.369 | 0.160 | 27.067 | 14.216 |
|  | *Tnf* | 13.442 | 39.245 | 8.386 | 0.914 |
|  | *Cd4* | 12.699 | 6.377 | 0.001 | 3.421 |
|  | *Il13* | 2.540 | 13.343 | 4.243 | 1.718 |
|  | *Cd8* | 1.058 | 5.691 | 16.902 | 0.948 |
|  | *Il10* | 33.766 | 1.204 | 0.018 | 2.809 |
|  | *Il6* | 21.846 | 2.361 | 33.435 | 0.059 |
|  | *Ifng* | 1.820 | 9.831 | 8.187 | 44.170 |

| **Myeloid BALB/c** | Importance of components: |  |  |  |  |
| --- | --- | --- | --- | --- | --- |
|  |  | PC1 | PC2 | PC3 | PC4 |
|  | Standard deviation | 1.473 | 1.003 | 0.598 | 0.000 |
|  | Proportion of Variance | 0.614 | 0.285 | 0.101 | 0.000 |
|  | Cumulative Proportion | 0.614 | 0.899 | 1.000 | 1.000 |
|  | Contribution |  |  |  |  |
|  |  | Dim.1 | Dim.2 | Dim.3 | Dim.4 |
|  | *Ptges* | 1.697 | 22.224 | 1.238 | 20.611 |
|  | *Cd206* | 4.447 | 0.233 | 13.326 | 4.409 |
|  | *Cd68* | 7.060 | 0.843 | 11.294 | 11.058 |
|  | *Il4r* | 0.106 | 2.742 | 8.906 | 25.094 |
|  | *S100a9* | 9.288 | 32.401 | 15.551 | 0.661 |
|  | *Cybb* | 8.481 | 4.672 | 9.611 | 0.373 |
|  | *Irg1* | 55.007 | 0.009 | 0.162 | 4.568 |
|  | *Arg1* | 11.112 | 3.127 | 20.797 | 7.582 |
|  | *Nos2* | 2.803 | 33.749 | 19.116 | 25.645 |

| **Lymphoid *Slamf1^-/-^*** | Importance of components: |  |  |  |  |
| --- | --- | --- | --- | --- | --- |
|  |  | PC1 | PC2 | PC3 | PC4 |
|  | Standard deviation | 1.410 | 1.068 | 0.737 | 0.000 |
|  | Proportion of Variance | 0.541 | 0.311 | 0.148 | 0.000 |
|  | Cumulative Proportion | 0.541 | 0.852 | 1.000 | 1.000 |
|  | Contributions |  |  |  |  |
|  |  | Dim.1 | Dim.2 | Dim.3 | Dim.4 |
|  | *Foxp3* | 0.026 | 1.714 | 10.788 | 1.680 |
|  | *Il17* | 21.944 | 9.200 | 5.354 | 9.642 |
|  | *Tgfb* | 0.451 | 5.365 | 9.412 | 30.954 |
|  | *Tnf* | 28.222 | 19.056 | 10.762 | 2.744 |
|  | *Cd4* | 2.105 | 2.317 | 1.538 | 0.239 |
|  | *Il13* | 0.355 | 2.656 | 18.473 | 4.629 |
|  | *Cd8* | 41.183 | 35.248 | 2.182 | 1.569 |
|  | *Il10* | 3.410 | 7.951 | 1.737 | 38.838 |
|  | *Il6* | 0.666 | 11.768 | 34.429 | 5.439 |
|  | *Ifng* | 1.637 | 4.724 | 5.324 | 4.265 |

| **Myeloid *Slamf1^-/-^*** | Importance of components: |  |  |  |  |
| --- | --- | --- | --- | --- | --- |
|  |  | PC1 | PC2 | PC3 | PC4 |
|  | Standard deviation | 1.416 | 0.824 | 0.534 | 0.000 |
|  | Proportion of Variance | 0.676 | 0.228 | 0.096 | 0.000 |
|  | Cumulative Proportion | 0.676 | 0.904 | 1.000 | 1.000 |
|  | Contributions |  |  |  |  |
|  |  | Dim.1 | Dim.2 | Dim.3 | Dim.4 |
|  | *Ptges* | 1.345 | 1.358 | 1.671 | 2.503 |
|  | *Cd206* | 0.108 | 0.203 | 26.834 | 2.283 |
|  | *Cd68* | 0.648 | 5.761 | 8.430 | 30.260 |
|  | *Il4r* | 0.253 | 3.018 | 1.462 | 20.834 |
|  | *S100a9* | 6.399 | 14.679 | 18.406 | 1.540 |
|  | *Cybb* | 2.066 | 5.664 | 13.352 | 28.399 |
|  | *Irg1* | 51.200 | 1.100 | 15.090 | 9.935 |
|  | *Arg1* | 37.965 | 0.591 | 14.750 | 4.194 |
|  | *Nos2* | 0.015 | 67.626 | 0.006 | 0.051 |
