# Supplementary figures and images for: Interaction of Signaling Lymphocytic Activation Molecule Family 1 (SLAMF1) receptor with Trypanosoma cruzi is strain-dependent and affects NADPH oxidase expression and activity
Source: PLoS Negl Trop Dis. 2020 Sep 14;14(9):e0008608. doi: 10.1371/journal.pntd.0008608 (PMC7515593; doi:10.1371/journal.pntd.0008608)

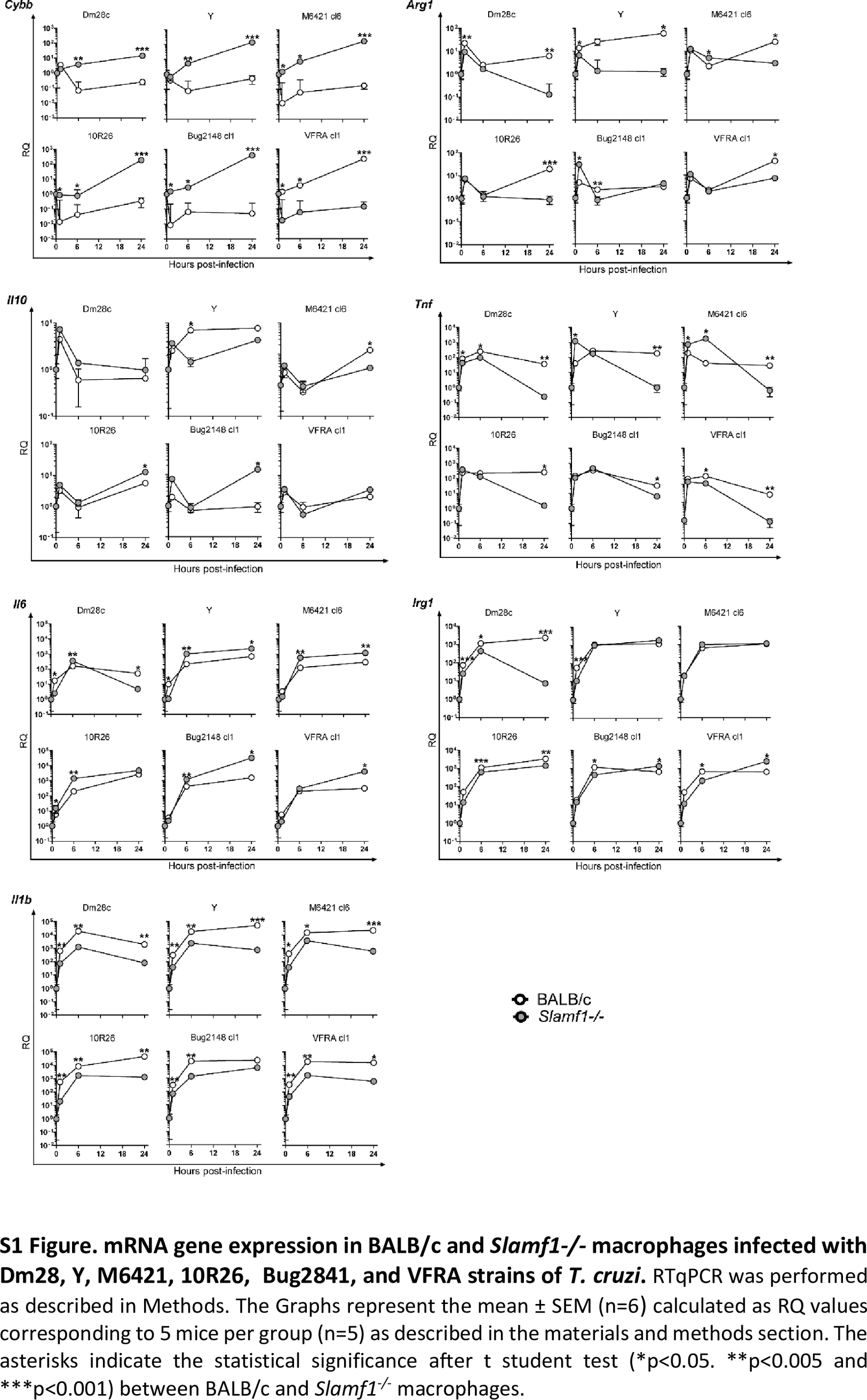

Supplement: S1 Fig — RTqPCR was performed as described in Methods. The Graphs represent the mean ± SEM (n = 6) calculated as RQ values corresponding to 5 mice per group (n = 5) as described in the materials and methods section. The asterisks indicate the statistical significance after t student test (*p<0.05. **p<0.005 and ***p<0.001) between BALB/c and Slamf1-/- macrophages. (TIF) [file pntd.0008608.s007.tif]

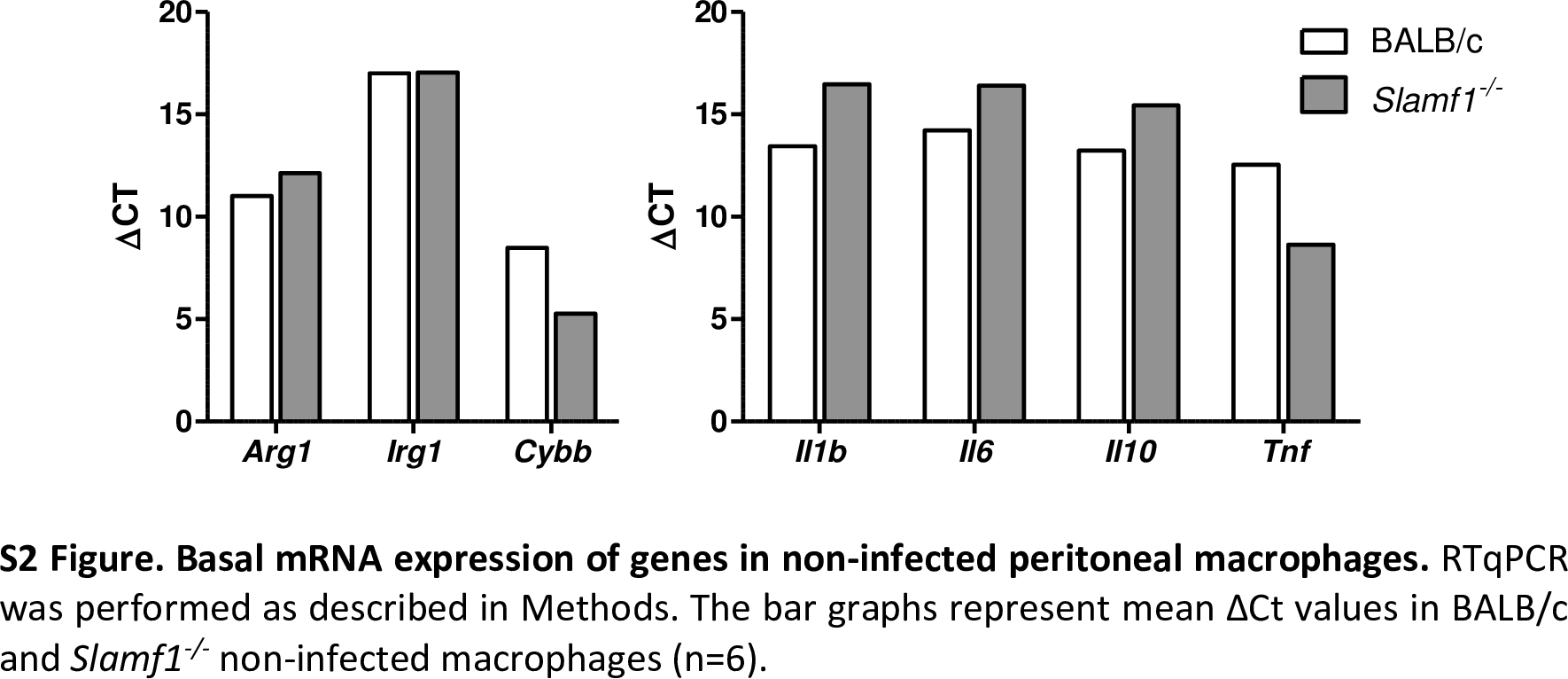

Supplement: S2 Fig — RTqPCR was performed as described in Methods. The bar graphs represent mean ΔCt values in BALB/c and Slamf1-/- non-infected macrophages (n = 6). (TIF) [file pntd.0008608.s008.tif]

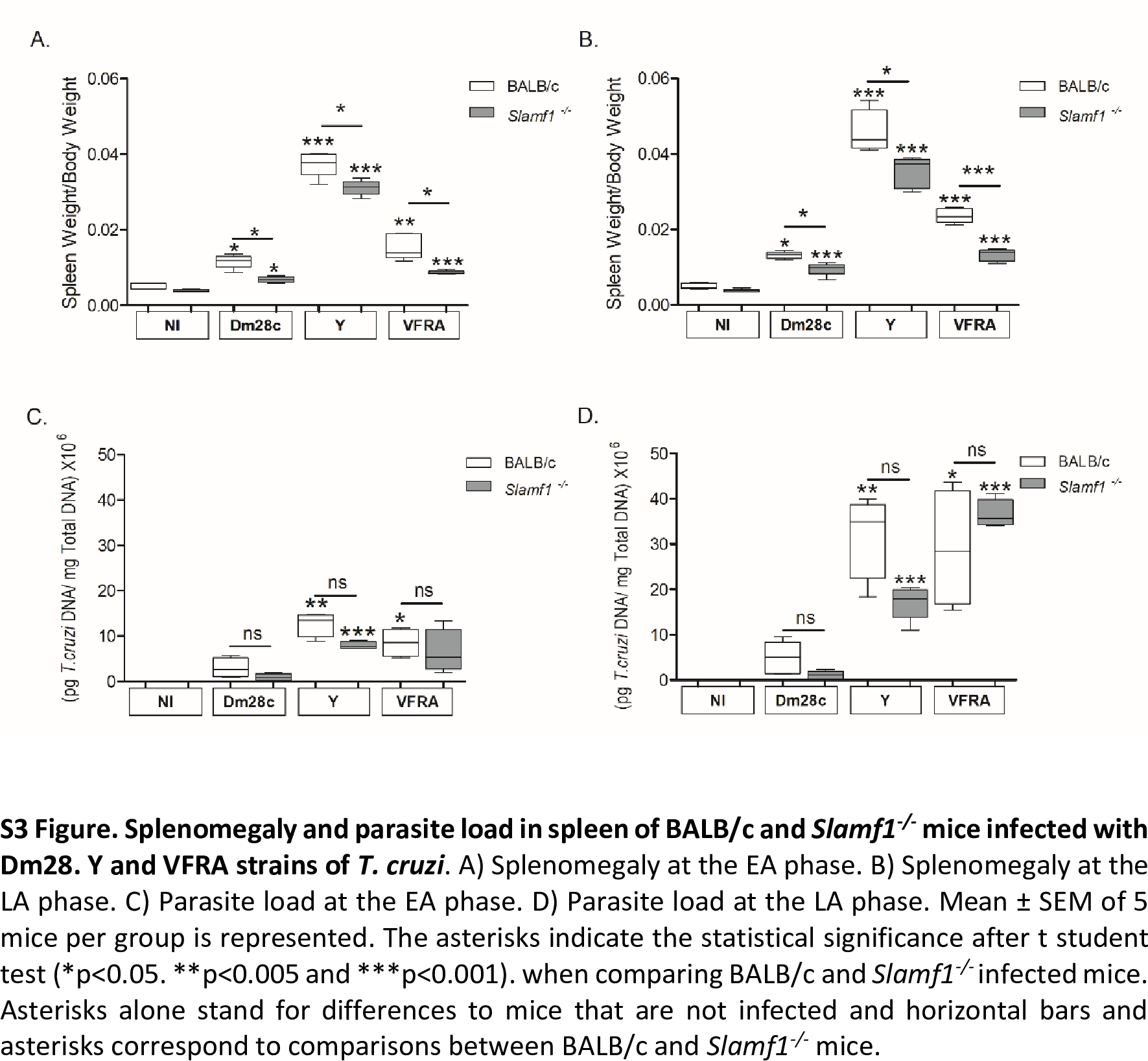

Supplement: S3 Fig — A) Splenomegaly at the EA phase. B) Splenomegaly at the LA phase. C) Parasite load at the EA phase. D) Parasite load at the LA phase. Mean ± SEM of 5 mice per group is represented. The asterisks indicate the statistical significance after t student test (*p<0.05. **p<0.005 and ***p<0.001). when comparing BALB/c and Slamf1-/- infected mice. Asterisks alone stand for differences to mice that are not infected and horizontal bars and asterisks correspond to comparisons between BALB/c and Slamf1-/- mice. (TIF) [file pntd.0008608.s009.tif]

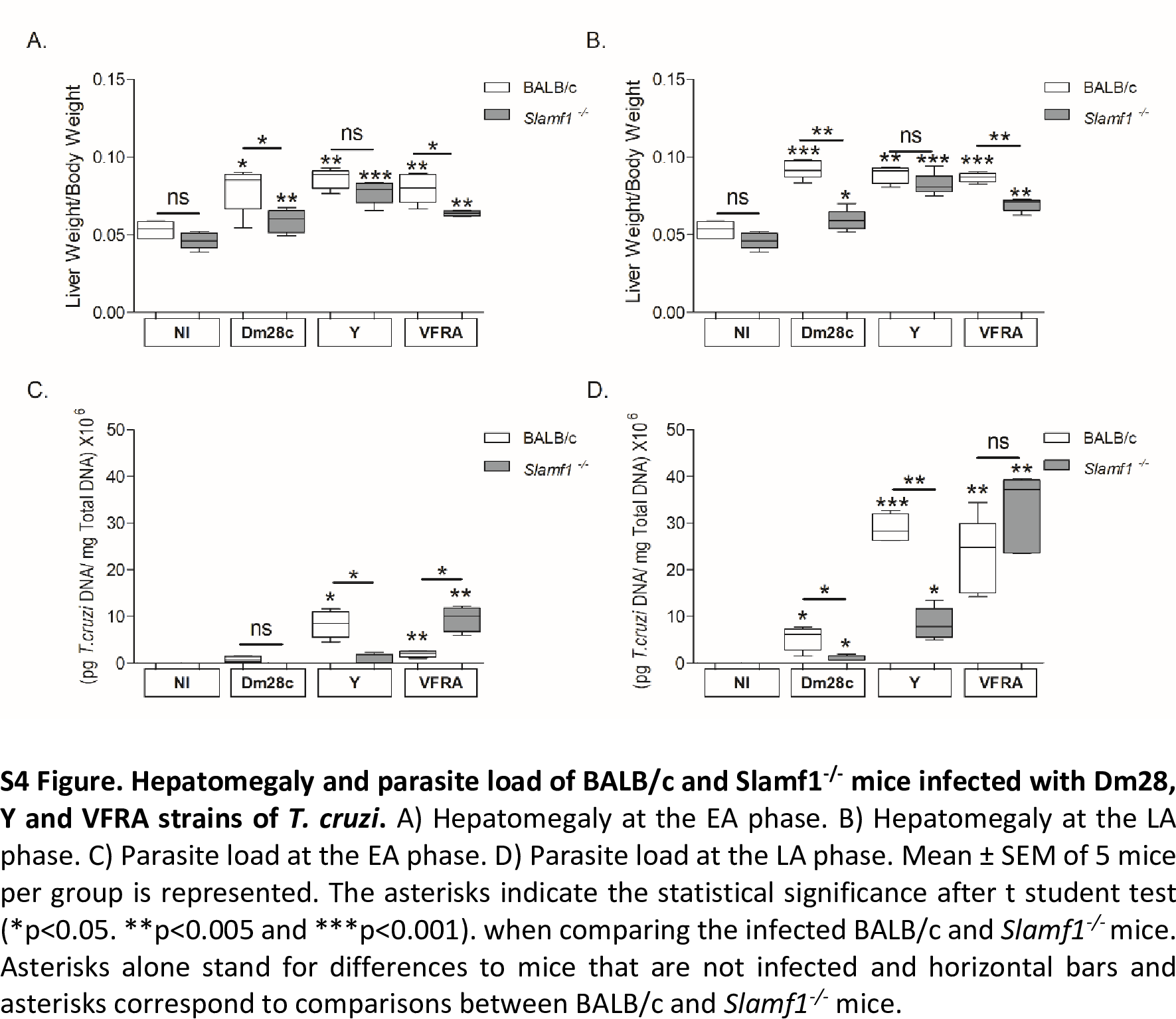

Supplement: S4 Fig — A) Hepatomegaly at the EA phase. B) Hepatomegaly at the LA phase. C) Parasite load at the EA phase. D) Parasite load at the LA phase. Mean ± SEM of 5 mice per group is represented. The asterisks indicate the statistical significance after t student test (*p<0.05. **p<0.005 and ***p<0.001). when comparing the infected BALB/c and Slamf1-/- mice. Asterisks alone stand for differences to mice that are not infected and horizontal bars and asterisks correspond to comparisons between BALB/c and Slamf1-/- mice. (TIF) [file pntd.0008608.s010.tif]

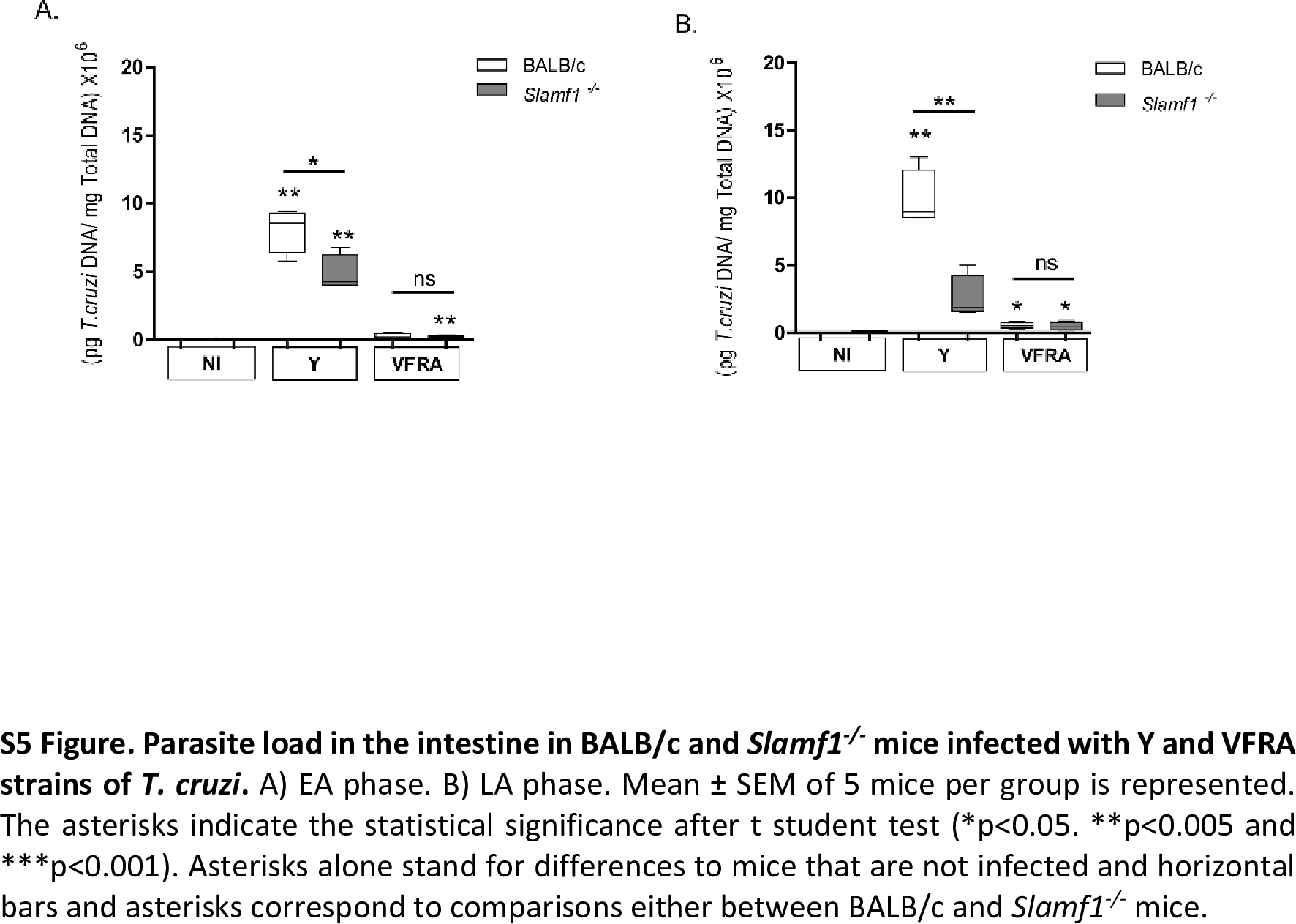

Supplement: S5 Fig — A) EA phase. B) LA phase. Mean ± SEM of 5 mice per group is represented. The asterisks indicate the statistical significance after t student test (*p<0.05. **p<0.005 and ***p<0.001). Asterisks alone stand for differences to mice that are not infected and horizontal bars and asterisks correspond to comparisons either between BALB/c and Slamf1-/- mice. (TIF) [file pntd.0008608.s011.tif]

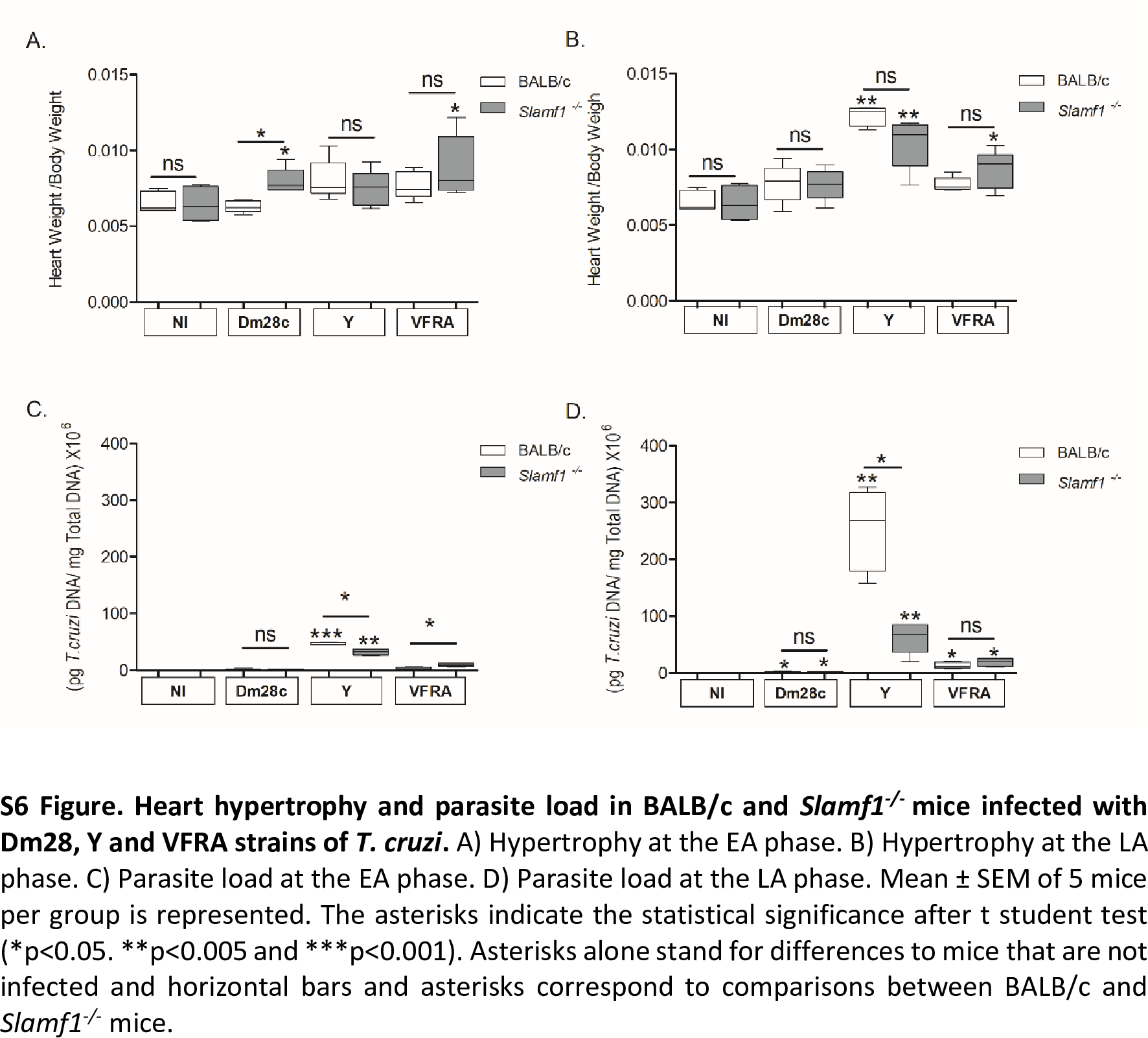

Supplement: S6 Fig — A) Hypertrophy at the EA phase. B) Hypertrophy at the LA phase. C) Parasite load at the EA phase. D) Parasite load at the LA phase. Mean ± SEM of 5 mice per group is represented. The asterisks indicate the statistical significance after t student test (*p<0.05. **p<0.005 and ***p<0.001). Asterisks alone stand for differences to mice that are not infected and horizontal bars and asterisks correspond to comparisons between BALB/c and Slamf1-/- mice. (TIF) [file pntd.0008608.s012.tif]

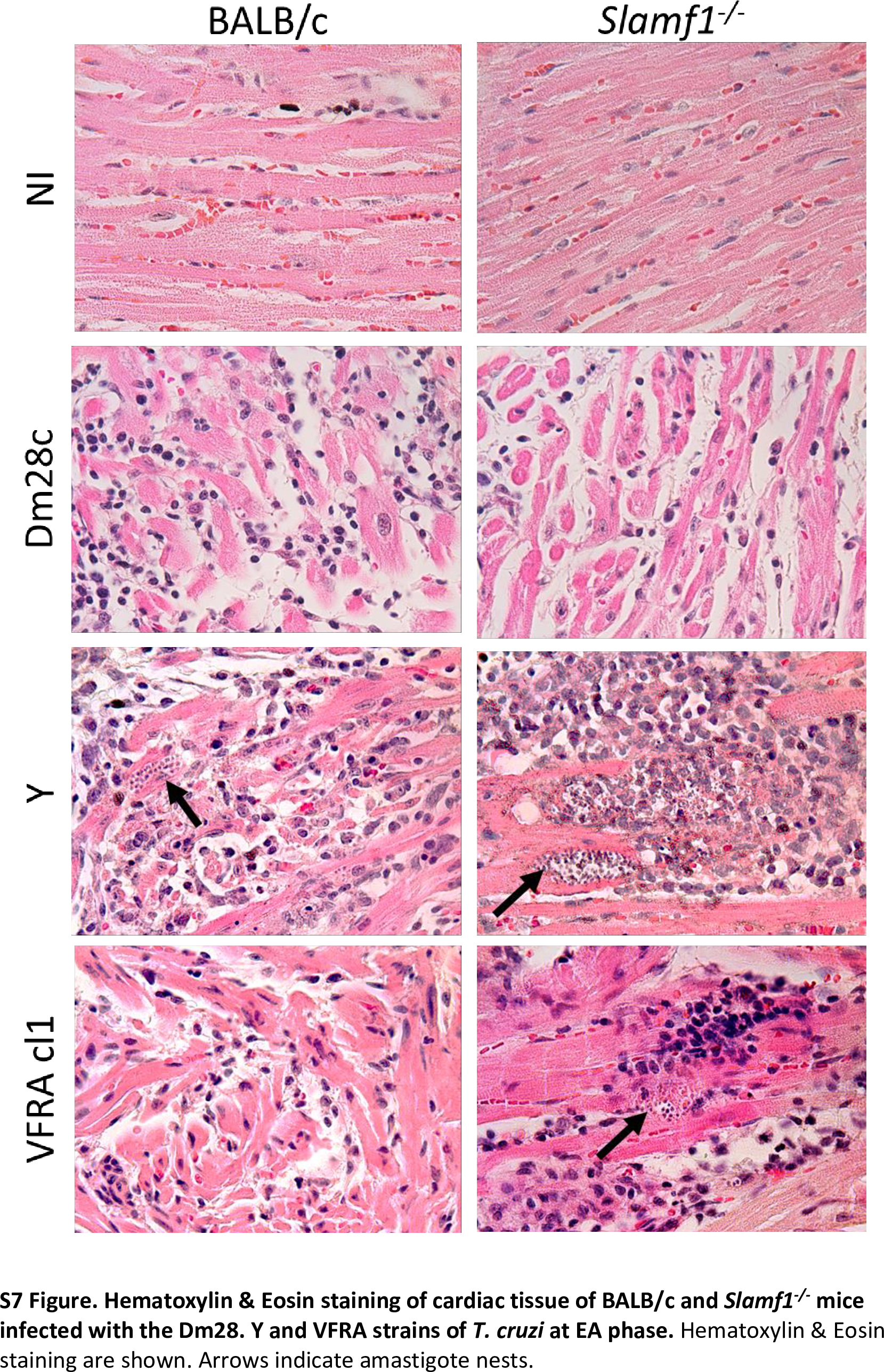

Supplement: S7 Fig — Y and VFRA strains of T. cruzi at EA phase. Hematoxylin & Eosin staining are shown. Arrows indicate amastigote nests. (TIF) [file pntd.0008608.s013.tif]

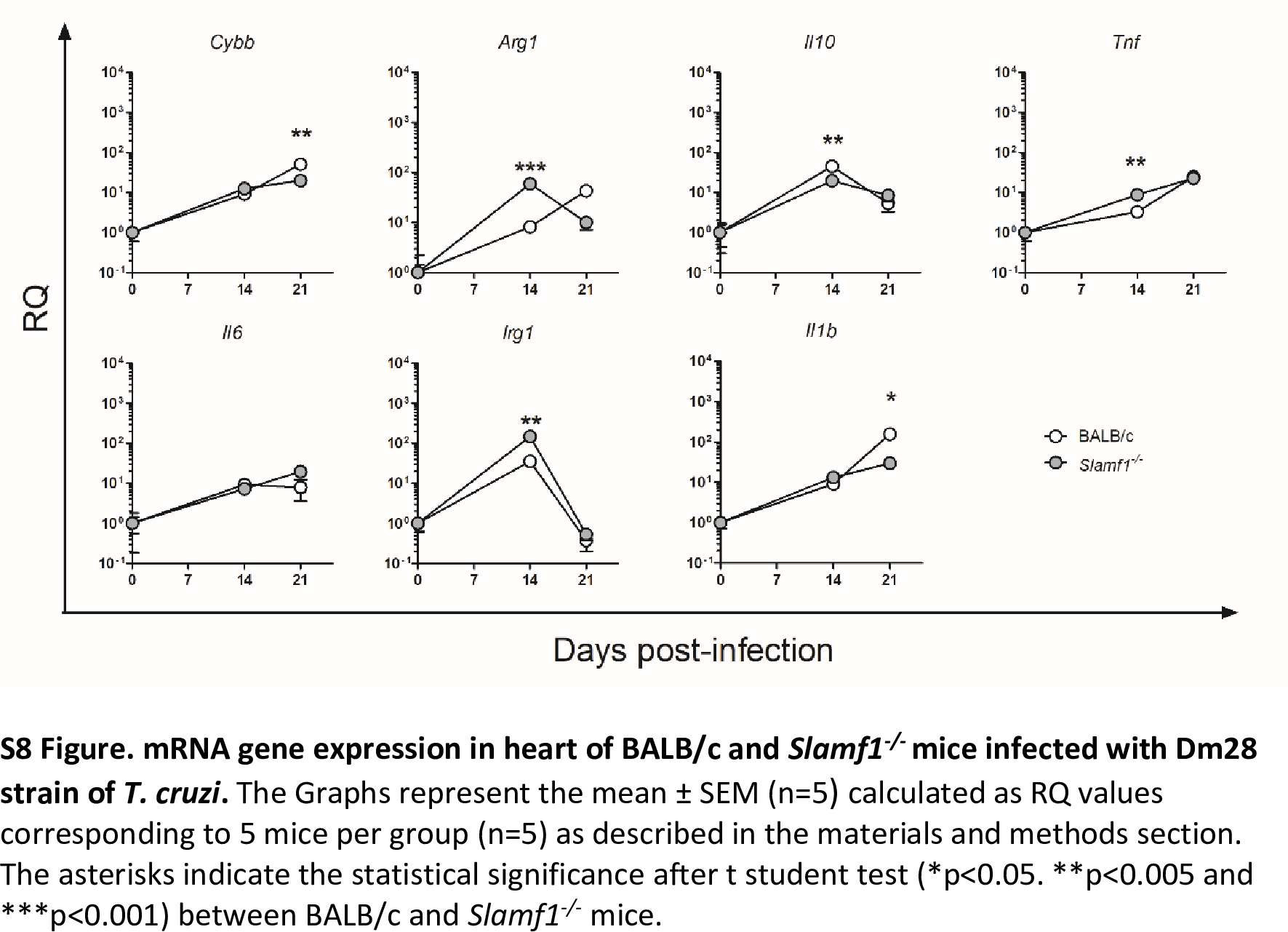

Supplement: S8 Fig — The Graphs represent the mean ± SEM (n = 5) calculated as RQ values corresponding to 5 mice per group (n = 5) as described in the materials and methods section. The asterisks indicate the statistical significance after t student test (*p<0.05. **p<0.005 and ***p<0.001) between BALB/c and Slamf1-/- mice. (TIF) [file pntd.0008608.s014.tif]
